# A broad survey reveals substitution tolerance of residues ligating FeS clusters in [NiFe] hydrogenase

**DOI:** 10.1186/1471-2091-15-10

**Published:** 2014-06-17

**Authors:** Isaac T Yonemoto, Benjamin R Clarkson, Hamilton O Smith, Philip D Weyman

**Affiliations:** 1 Department of Synthetic Biology and Bioenergy, J. Craig Venter Institute, 4120 Capricorn Lane, La Jolla, CA 92037, USA

**Keywords:** [NiFe] hydrogenase, Iron-sulfur cluster, *Alteromonas macleodii* deep ecotype

## Abstract

**Background:**

In order to understand the effects of FeS cluster attachment in [NiFe] hydrogenase, we undertook a study to substitute all 12 amino acid positions normally ligating the three FeS clusters in the hydrogenase small subunit. Using the hydrogenase from *Alteromonas macleodii* “deep ecotype” as a model, we substituted one of four amino acids (Asp, His, Asn, Gln) at each of the 12 ligating positions because these amino acids are alternative coordinating residues in otherwise conserved-cysteine positions found in a broad survey of NiFe hydrogenase sequences. We also hoped to discover an enzyme with elevated hydrogen evolution activity relative to a previously reported “G1” (H230C/P285C) improved enzyme in which the medial FeS cluster Pro and the distal FeS cluster His were each substituted for Cys.

**Results:**

Among all the substitutions screened, aspartic acid substitutions were generally well-tolerated, and examination suggests that the observed deficiency in enzyme activity may be largely due to misprocessing of the small subunit of the enzyme. Alignment of hydrogenase sequences from sequence databases revealed many rare substitutions; the five substitutions present in databases that we tested all exhibited measurable hydrogen evolution activity. Select substitutions were purified and tested, supporting the results of the screening assay. Analysis of these results confirms the importance of small subunit processing. Normalizing activity to quantity of mature small subunit, indicative of total enzyme maturation, weakly suggests an improvement over the “G1” enzyme.

**Conclusions:**

We have comprehensively screened 48 amino acid substitutions of the hydrogenase from *A. macleodii* “deep ecotype”, to understand non-canonical ligations of amino acids to FeS clusters and to improve hydrogen evolution activity of this class of hydrogenase. Our studies show that non-canonical ligations can be functional and also suggests a new limiting factor in the production of active enzyme.

## Background

The development of hydrogen as a biofuel is appealing because unlike hydrocarbon-based biofuels, the production of hydrogen involves very few chemical steps, and thus may be more efficient. Ultimately, one goal is to couple biohydrogen production to the photosynthetic apparatus to directly and efficiently capture solar energy. Hydrogen-producing redox enzymes tend to be highly oxygen sensitive [1-3], and the first stage of photosynthesis is the production of molecular oxygen. Using a [NiFe] hydrogenase with some degree of oxygen tolerance, we elected a strategy of improving the enzyme activity by investigating alternative amino acid ligation of hydrogenase FeS clusters [4].

The broad family of two-subunit [NiFe] hydrogenases, including [NiFeSe] hydrogenases, have many non-cysteinyl amino acid ligands for FeS clusters. An alignment of predicted [NiFe] hydrogenase amino acid sequences from sequenced genomes in the NCBI GenBank database revealed several ‘unusual’ amino acids in positions normally containing cysteine and known to ligate Fe-S clusters involved in electron transport to the active site (Table 1). Previous researchers have heterologously expressed a small subunit containing unusual Fe-S ligations and have verified the presence of intact clusters of the expected 4Fe4S type in spite of these unusual amino acids [5], adding confidence that the amino acids predicted at these sites are not the result of sequencing errors. We sought to use our robust *Alteromonas macleodii* heterologous expression system [4,6-8] to conduct a preliminary examination broadly surveying these and similar substitutions, both to find a better performing enzyme and to launch interest in better understanding FeS clusters.

**Table 1:** FeS cluster substitutions observed in sequence databases

**Species**	**Accession**	**Cluster, position**	**Substitution**
*M. barkeri*	YP_305362	Proximal (4Fe4S)?, 1	Cys to Asp
*G. metalloreducens*	YP_006722286	Proximal (4Fe4S)?, 2	Cys to Asp
*N. punctiforme*	YP_001864093	Proximal (4Fe4S), 2	Cys to Asn
*N. punctiforme*	YP_001864093	Distal (4Fe4S), 1	His to Gln
*N. punctiforme*	YP_001864093	Medial (3Fe4S), 2	Pro to Lys*
*D. baculatum*	YP_003157302	Medial (4Fe4S), 2	Pro to Cys

Putative FeS cluster ligating residues deviating from the standard pattern seen among two-subunit [NiFe]-hydrogenases. Each substitution is represented by multiple members; only one representative example is listed. Question marks indicate FeS cluster assignments that have not been confirmed. Asterisk (“*”) indicates a substitution not investigated in this study. The final entry represents [NiFeSe] hydrogenases, which do feature a cysteine in lieu of a proline.

Typically, experiments substituting Fe-S ligation sites substitute ligating cysteines with serines, which are the most structurally homologous to cysteine [9-12], although histidinyl ligations have also been substituted [13]. However, in nature, serinyl FeS cluster ligation is, to date, unknown, although there is at least one example of a threoninyl FeS ligation [14]. In our study, we sought to substitute these ligating cysteines to a panel of amino acid residues suspected to ligate FeS clusters in hydrogenases. Therefore, we permuted all of the amino acids in the set {Asp, His, Asn, Gln}, which are found in various subclasses of two-subunit hydrogenase (Table 1), at sites containing ligating cysteines. Although glutamic acid and lysine are also found in ligating positions of two-subunit [NiFe] hydrogenases, we chose to avoid these residues. Glutamic acid was avoided because of its paucity in hydrogenase alignments at conserved cysteine positions, and its structural change relative to cysteine. Lysine was not used because of its even more exaggerated structural difference. Accommodating either of these residues would likely require additional second-sphere residue substitutions to adjust the structure of the hydrogenase.

We also surmised that residue substitutions may alter the electronic landscape of the FeS cluster chain in hydrogenase to allow for improved electron flow. Following the precepts of Marcus theory, an ideal electron transfer chain for hydrogen production would feature a modestly energetically downhill (increasing midpoint potential) series of FeS clusters proceeding from the distal site and leading to the active site [15]. Instead, most two-subunit hydrogenases feature a 3Fe4S, high potential medial cluster [16,17]. Previous work has shown a modest increase in hydrogen production upon converting this hydrogenase to a 4Fe4S cluster with a corresponding lower midpoint potential, and although closer, the midpoint potential is still not tuned to an optimal range [17]. Although we do not yet have concrete proof that the homologous substitution in the *A. macleodii* hydrogenase results in a 4Fe4S cluster, nor do we have midpoint potential measurements, we do observe a marked increase in hydrogen production for our first generation “G1” (H230C/P285C^a^) engineered hydrogenase in which the medial FeS cluster Pro and the distal FeS cluster His were each substituted for Cys [8]. Proceeding from this point, we hoped our comprehensive substitution study could serve as an exercise to screen for a further improved enzyme.

In this work, we screened the activity of 48 ligation variants using a crude whole cell hydrogen evolution assay. The whole cell assay revealed two interesting facts: First, that all substitutions homologous to those observed in the pan-hydrogenase family were tolerated enough to yield measurably active enzyme. Secondly, cysteine-to-aspartic acid substitutions as a class were generally well-tolerated (with one exception). Aspartate-ligated FeS clusters have been observed in the natural case of *Pyrococcus* ferredoxin; this non-canonical ligation results in a 4Fe4S cluster with an aerobically labile Fe atom [18-20]. In the case of the photosystem protein PsaC, artificial substitution can result in the generation of a 3Fe4S cluster in lieu of a 4Fe4S cluster [10,21], but one Cys-Asp substitution resulted in a stable 4Fe4S cluster with a decreased redox potential [22]. In our *A. macleodii* hydrogenase substitution set, further examination of 8 aspartic acid variants suggested that activity differences resulted from enzyme misprocessing, observed as a lack of small subunit N-terminal cleavage. Careful isolation of the enzyme alone suggested that two of these aspartic acid-substituted enzymes may be intrinsically more efficient at hydrogen evolution than the G1 enzyme upon which they are based.

## Results and discussion

### Hydrogenase screening assay

**Figure 1:**
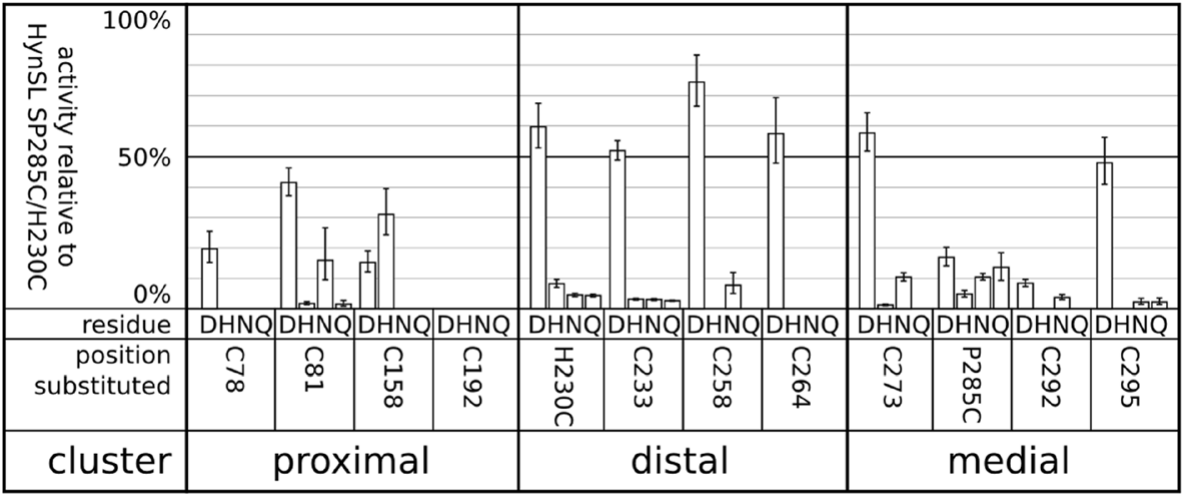
**Crude whole cell screening assay for hydrogen evolution activity.** Relative activities of amino acid substitutions at each of the 12 substitution positions predicted to ligate Fe-S clusters are presented. Error bars represent geometric standard errors propagated through activity normalization.

We constructed 48 variants of hydrogenase featuring substitutions from the targeted set of four amino acids in the background of our first generation “G1” (H230C/P285C^a^) engineered hydrogenase. The G1 strain has the medial FeS cluster proline to cysteine substitution and the distal FeS cluster histidine to cysteine substitution [8]. Thus, in the G1 starter strain, cysteine was present in all 12 coordinating positions, and each of the 48 variants substituted one of four amino acids (Asp, His, Asn, Gln) at one of the 12 positions.When these variants were tested in a crude whole cell activity assay (Figure 1), several trends emerged. First, aspartic acid was almost always the most tolerated substitution. In addition to the P285C substitution found in [NiFeSe] hydrogenases, which is present in our chosen background sequence, all substitutions homologous to those found in known sequences were also functional (C78D, C81D/N, H230Q). Finally, there appeared to be “privileged” ligations (C78, C192, C264) wherein no substitutions or only the aspartic acid substitution resulted in detectable activity, suggesting a higher level of sensitivity to substitution at these positions.

### Bacterial lysate hydrogen evolution activity assay

**Figure 2:**
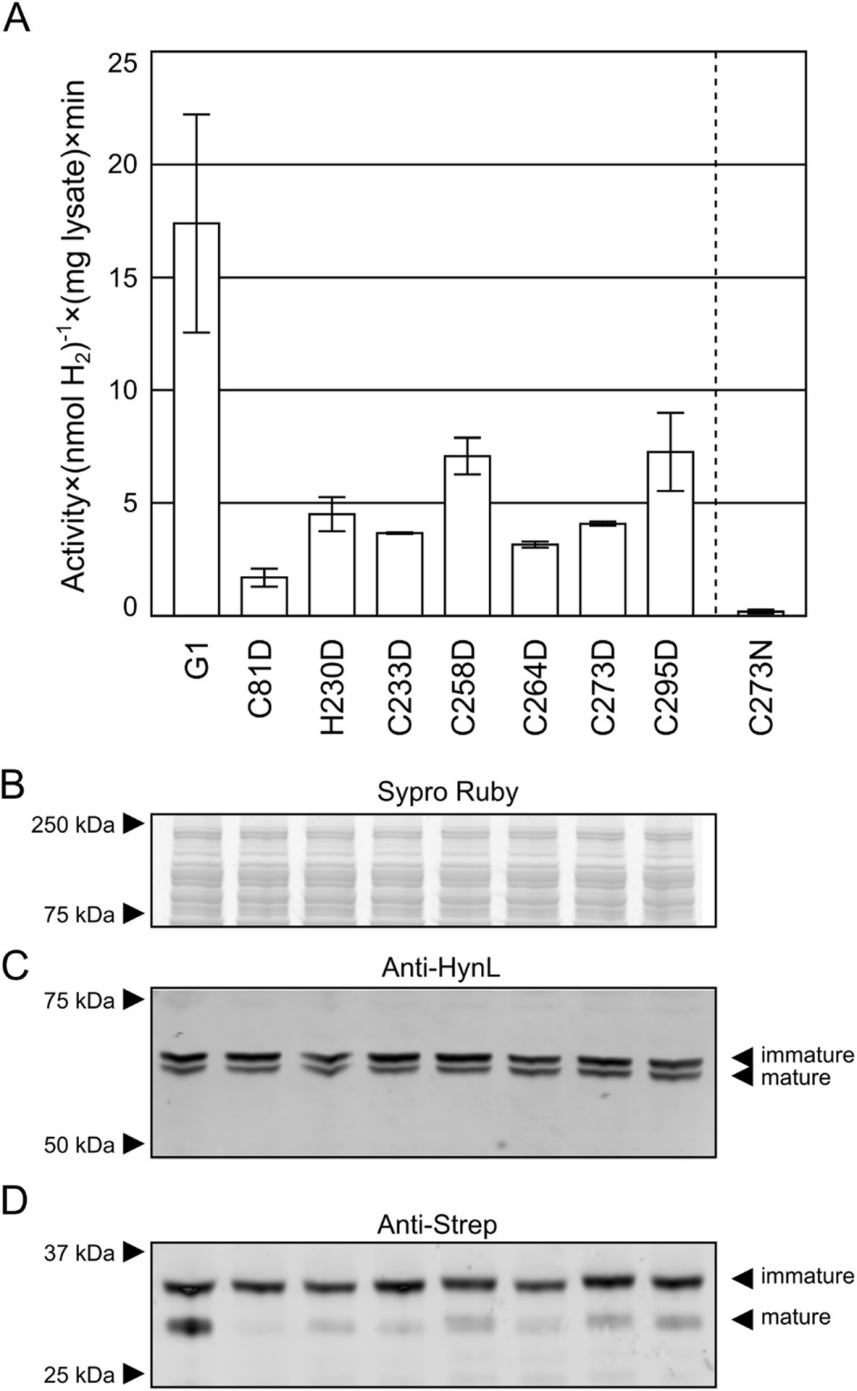
**Bacterial lysate activity assay. A)***Hydrogen evolution* activities of hydrogenases bearing select substitutions as ascertained by by bacterial lysate assay. Error bars represent the standard error of the mean activity. The dotted line separates Asp substitution data from Asn substitution data. **B)** SYPRO Ruby stain loading control for **C)** anti-HynL and **D)** anti-Strep western blots of bacterial lysates of select substitutions demonstrating approximate enzyme yield.

To confirm the qualitative validity of the whole cell screening assay, we subjected the best-performing aspartic acid substitutions and one asparagine substitution to a bacterial lysate assay similar to that which we performed in our initial development of the G1 enzyme [8]. Here the results qualitatively tracked the results observed from the initial screening experimental set (Figure 2A). Western blot analysis of the lysates (Figure 2B, C, D), suggested that the enzyme yield roughly tracks small subunit processing efficiency. The immature (~69 kDa) and mature (~67 kDa) large subunit bands appear roughly unchanged. There are two small subunit bands, which we believe correspond to immature upper band (~36 kDa theoretical), and mature lower band (31 kDa theoretical), corresponding to cleavage by the twin arginine translocon (TAT) machinery in the sequence ATA/LGN (residues 52–57). This cleavage only occurs after the entire enzyme is properly assembled and exported via the TAT system – although in the case of the *A. macleodii* hydrogenase, the hydrogenase likely returns to the cytoplasm [4].Of these two small subunit bands, the immature fraction appears to have little correlation with the activity, but qualitatively, the mature fraction strongly correlates with the observed activities (Figure 2D). This could be for various reasons, such as a kinetic barrier to loading the FeS cluster preventing assembly, or a downstream effect such as clearance of unstable enzyme variants by proteolysis. These results encouraged us to conduct further study on the “best of class” aspartic acid residues to separate the enzyme intrinsic activity from these cell-dependent extrinsic factors.

### Doubly substituted enzyme assay

Using a pooled Gibson isothermal assembly strategy, we generated a comprehensive library of double substitutions (see Additional file 1: Table S1) drawing from a set of distal and medial cluster substitutions (8 total constructs discovered from a pool of 16). This library also contained a handful of unplanned, spontaneous triple substitutions of unresolved assembly mechanism. No multiply-substituted construct tested yielded detectable activity, and based on our observations following western blots of other constructs, we suspect that maturation of the hydrogenase is too compromised in the multiple substitutions.

### Tandem purification activity assay

**Figure 3:**
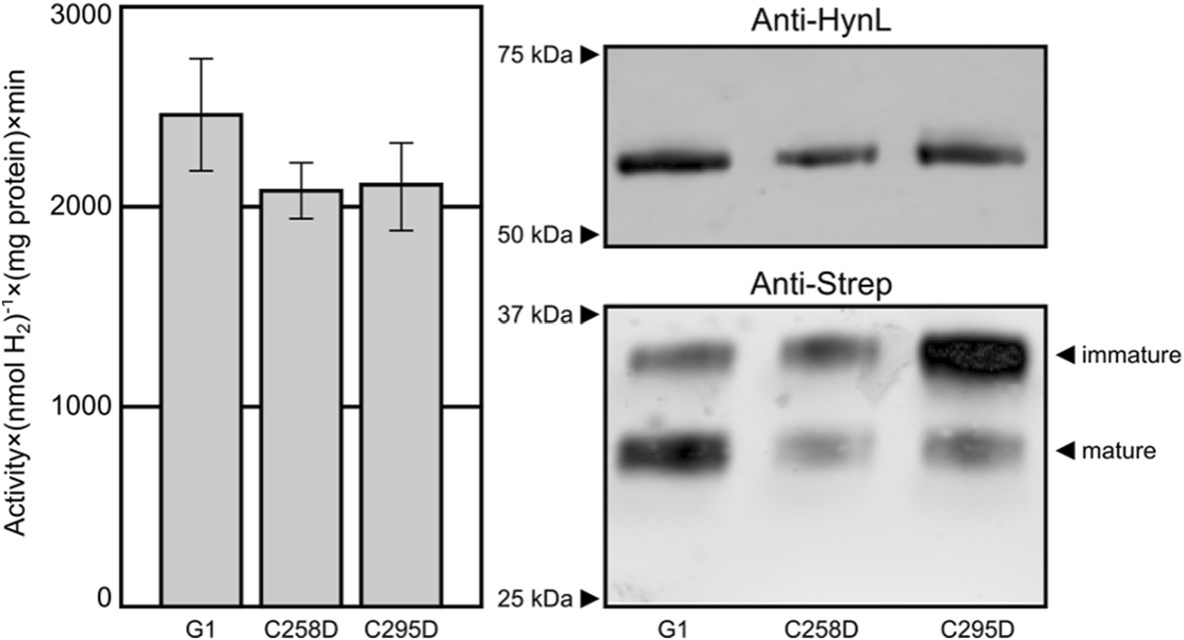
**Purified protein activity assay.** Left: Hydrogen evolution activities of G1, C258D, and C295D after purification using IMAC/strep-tactin tandem purification. Error bars represent the standard error of the mean activity. Right: anti-HynL and anti-strep western blots of enzyme samples.

We subjected the G1 enzyme and the two best-performing variants (C258D, C295D) to a tandem purification assay to better understand the intrinsic activity of the enzyme, removed from complicating factors of the enzyme maturation process. The tandem purification appears to remove nearly all contaminating proteins, at least as detectable by SYPRO Ruby-stained SDS-PAGE gels, and generates well-resolved bands that appear to correspond to the large and small subunit (see Additional file 1: Figure S1). After normalizing to protein content, we observed little difference in specific activity between the three variants (Figure 3, left). Western blot analysis shows (Figure 3, right) the unit composed of the processed large subunit and unprocessed small subunit species (P-U) is a dominant portion of the total enzyme for both the C258D and C295D variants suggesting an extrinsic processing or clearance defect that results from these substitutions. SYPRO Ruby staining shows a qualitatively similar pattern, suggesting that the observation is not an artefact of antibody binding (Additional file 1: Figure S1). A TAT-signal processing defective variant (substituting the twin arginine motif for a twin lysine residues) had no detectable activity (Additional file 1: Table S2, pIY009) suggesting that this P-U species represents an inactive fraction of the total enzyme. Densitometric analysis (n = 2) was used to quantify the processed small subunit bands, and normalization of hydrogen evolution activity to these adjusted values based on the active band suggests that C258D is approximately 4 × more active than G1 and C295D is approximately 2 × more active than G1. Western blot densities of HynL bands correlated to total protein, further suggesting the purification procedure did not discriminate between P-U and fully processed enzyme.

## Conclusions

The first objective of this study was to investigate broadly whether hydrogenases tolerate the unusual amino acid residues at the ligating positions in their primary structure. Our discovery was that the *A. macleodii* hydrogenase is broadly tolerant of cysteine substitutions from the set {Asp, His, Asn, Gln}; each of these residues yielded activity when substituted at some position. Although some position/residue combinations were not tolerated, all substitutions homologous to substitutions observed in the sequence databases yielded measurable hydrogen evolution activity. Our screening assay only tested the evolution direction, so it is also possible that even among those substitutions that are unmeasurably poor in our assay, certain substitutions may remain active in the uptake direction.

We further discovered that the aspartic acid substitutions were the most active among all of the tested substitutions, likely because of a minimal structural perturbation relative to cysteine (shared with asparagine) and shared electronegative quality upon ligation to an FeS cluster. Examination of the lysates further suggested that the dominant reason for the loss of activity relative to the G1 enzyme likely results from a failure to properly process the N-terminal TAT signal; further experiments may need to be conducted to properly assess this effect, possibly by tagging and separating the two dimeric species. Although all the motifs we tested were active, their lowered activities suggests that efficiency of small subunit synthesis may depend on either second-sphere residues participating in the installation of FeS clusters, or alternatively, host FeS cluster synthesis machinery capable of installing FeS clusters around different motifs.

The second objective of this study was to discover hydrogenases that are more active in the evolution direction, possibly achieving this by modulating the electronic landscape of the FeS pathway to the active site. Normalization of hydrogen evolution to only the variant of the small subunit that is likely active suggests that the G1/C258D substitution is approximately 4 times more active than the G1 enzyme, although in order to fully take advantage of this improvement, a corresponding improvement in extrinsic processing of the enzyme would need to be developed. The *isc*-related ferredoxin *fdx* is known to be involved in the small subunit processing of the native *E. coli* hydrogenases [23], and overexpression of the *isc* cluster has been used to improve yield of cyanobacterial ferredoxins [24]. A similar strategy of tandem overexpression of the native *E. coli isc* or *suf* clusters, or heterologous expression of the *A. macleodii suf* cluster may achieve this goal. Orthogonal strategies to improve electron transport through these FeS clusters may involve manipulation of second-sphere amino acids to alter the electrostatic environment. Our results may be consistent with observations that the distal FeS cluster tunes hydrogen evolution activity in a pH dependent manner when the wild-type histidinyl ligation is present [25].

This study represents a starting point for further study into the nature of ‘non-canonical’ amino acid substitutions that attach to FeS clusters. Our expression system, with fairly minimal effort, enables isolation of highly purified hydrogenase making it a good candidate for such studies. Future studies should also consider mechanisms featuring alternative electron transfer pathways, as dye-mediated electron transfer may introduce artefactual effects, such as delivery to clusters other than the distal cluster. Finally, hydrogenases featuring other unusual FeS clusters which are ligated by lysines, or an unusual, recently discovered 4Fe3S cluster [26-28], may also be a target for comprehensive substitution to {Asp, His, Asn, Gln} in the fashion of this experiment.

## Methods

### Molecular biology and plasmid construction

Plasmid pBC001 is a derivative of pIY107, which contains the *Alteromonas macleodii* “Deep ecotype” (DSM 17117) [29] hydrogenase operon driven by four TRC promoters (Additional file 1: Table S3 and Additional file 1: Figure S2). In pIY107, the C-terminus of HynS is modified with a strep-tag [8,30,31] and the N-terminus of HynL is modified with a His_6_-tag. Details of the construction of pIY107 will be published elsewhere, but we provide its sequence in the Additional file 1. Plasmid pBC001 is a further modification of pIY107 where a sequence containing an AvrII site within the “*orf2*” gene of pIY107 was silently mutated, and a new AvrII site was added immediately before the terminator-promoter unit preceding the *hynS* ORF. With unique AvrII and AgeI restriction sites flanking the *hynS* gene, this modification facilitated site-directed mutagenesis of *hynS*.

To construct pBC001, two amplicons of pIY107 were generated using Q5 PCR kit (New England Biolabs): An “upstream” amplicon spanning the region from the AvrII site in the middle of *orf2* to the pTRC cassette, and a “downstream” amplicon spanning the pTRC cassette to the AgeI site at the leading end of the *hynL* gene. The upstream amplicon was generated by primers BC000AvrF and BC001AvrR; the downstream amplicon was generated by primers BC002AvrF and IY171HynSR [8]; sequences and descriptions of primers are provided in Additional file 1: Table S4. A three-piece Gibson isothermal assembly was then performed using both amplicons and pIY107 doubly digested with AgeI and AvrII. The sequence of the inserted region of pBC001 was confirmed by Sanger DNA sequencing (Operon).

Plasmids pBC002 – pBC049 were generated using amplicons generated by forward primer BC002AvrF and a reverse primer bearing the appropriate DNA mutations to effect a substitution, as well as a complementary forward primer and the reverse primer IY171HynSR. A full list of primers used is provided in Additional file 1: Table S4. These amplicons were subjected to Gibson isothermal assembly with plasmid pBC001 doubly digested with the AvrII and AgeI restriction enzymes. *E. coli* transformation was initially performed in NEB-5α (New England Biolabs) strain but then switched to Epi300 (Clontech). The DNA sequences of the inserted regions spanning the *hynS* ORF were confirmed by Sanger DNA sequencing (Operon).

Doubly substituted construct plasmids were generated by amplification of regions bearing the 4 best-performing distal and 2 best-performing medial cluster aspartic acid substitutions, followed by adjustment to match concentration, and Gibson isothermal assembly as described above. Sixteen colonies of the assembly library were picked, screened, and sequenced, fortuitously resulting in a full sampling of all 8 combinations of substitutions (likelihood ~25%) and an additional 2 triply-substituted constructions (mechanism of assembly unknown).

### Crude whole cell hydrogenase screening assay

To quickly ascertain the effect of an amino acid substitution, a crude screen for hydrogen evolution activity was employed. After transforming the plasmid bearing the hydrogenase into *Escherichia coli* strain BL21ΔH_4_ cells [8,32] and overnight colony outgrowth, individual colonies were picked and used to inoculate 1.7 mL of autoinduction media [33] in sterile 10 mL scintillation vials sealed with sterile natural rubber septa (Aldrich). Cultures were grown overnight (~24 hours) at 30°C, 200 rpm rotation. Following growth, 0.1 mL of 40 mg mL^−1^ methyl viologen (Aldrich) and 0.1 mL of 0.5 M potassium phosphate solution, pH 7.0, and 0.01 mL of 10% (w/v) Triton X-100 (Aldrich) were anaerobically added from a nitrogen-sparged master solution. Finally, 0.1 mL of 2 M sodium dithionite was anaerobically added and the sealed vial was incubated for 2–4 hours at 30°C. Total hydrogen evolved was assessed using gas chromatography (6890 N, Agilent) using a Fused Silica Molsieve 5A column (CP7537, Varian) of 250 μL samples taken from the vial headspace. Activity was normalized to the activity of pBC001-bearing cultures prepared in parallel.

### Bacterial lysate activity assay

Bacterial lysate activity was measured as previously described [8], except experiments were performed in 10 mL vials; sparging was conducted under nitrogen; 250 μL samples were taken from the vial headspace; and different chromatography apparatus was used (6890 N, Agilent).

Briefly, bacteria were lysed using a probe sonicator (Bransonic), and 0.2 mL lysate, cleared by centrifugation (16,000 × g, 4°C), was added to a solution containing methyl viologen and potassium phosphate solutions in proportions as described above and were then sparged with nitrogen gas. Sodium dithionite was added in proportions as described above, and incubated for 2 hours at 30°C. Total hydrogen evolved was assessed using gas chromatography and activity was normalized to total protein content of the lysates as measured by Bradford assay.

### Tandem IMAC/strep-tactin hydrogenase preparation

Purified hydrogenases were expressed in 100 mL of autoinduction media; containing 0.5% α-lactose and 0.01% glucose; instead of lysis buffer, NP2 buffer (50 mM Na_3_PO_4_, 100 mM NaCl, 1 mM 2-mercaptoethanol, pH 7.0) was used; sonication was performed in two batches; and sonicated cell matter was centrifuged for 20 minutes instead of 10.

An IMAC spin column was prepared by applying 200 μL of TALON cobalt resin (Clontech) to an empty micro bio-spin column (Bio-Rad) and rinsing twice with deionized water and once with NP2 buffer. All spins except as noted were performed at 27 × g for 10 s at 4°C. The resulting cleared lysate was applied in two batches to the spin column; each batch was run through the column three times using 20 s spins. The columns were further washed using NP2 buffer (1 × 1 mL), NP2 buffer + 0.01% (w/v) SDS (1 × 1 mL), NP2 buffer + 0.05% (v/v) tween-20 (5 × 1 mL). Elution was achieved using three applications of 200 μL of LP2 buffer (50 mM Na_3_PO_4_, 100 mM NaCl, 1 mM 2-mercaptoethanol, pH 5.0). Each 100 mL expression was split into two batches processed in parallel, with the elutions combined afterwards.

pH exchange was conducted by applying IMAC eluate in three rounds (15 m at 13,000 × g, 4°C) to a 30 kDa microcon spin membrane (YM-30, Millipore) followed by one round after adding 500 μL of NP2 buffer. Retentate was collected by inverting the cartridge and centrifuging (3 m at 3,000 × g), and rinsing with an additional 500 μL of NP2 buffer.

Streptactin purification was conducted by applying this total retentate (~600 μL) to 100 μL of strep-tactin magnetic beads (Qiagen) in a 1.5 mL Eppendorf tube (Denville), followed by 1 h incubation with end-over-end agitation at 4°C. Beads were immobilized using magnetic separation and exchanged with NP2 buffer (1 mL) four times, and eluted using 100 μL NP2B (NP2 + 10 mM biotin), followed by a second round of 50 μL NP2B buffer. Protein content was determined by Bradford assay (Bio-Rad) and adjusted to 0.02 mg mL^−1^ for all samples. Hydrogenase assay was measured as above, except 20 μL of purified enzyme was diluted to 0.2 mL in NP2; and hydrogen evolution was conducted over 20 hours.

### SDS-PAGE analyses

Protein samples of crude, IMAC-purified, and tandem-purified samples were adjusted to matching protein content (0.1 mg mL^−1^, 0.1 mg mL^−1^, and 0.02 mg mL^−1^ respectively), supplemented with 5 × SDS-page loading buffer, and boiled for 5 minutes. These samples were then loaded onto a 10% NuPAGE Bis-Tris gel with the NuPAGE MOPS-SDS running buffer system (Invitrogen), and run on ice at 150 V for 2 or 4 hours.

For western blot analysis of crude samples from the “bacterial lysate assay”, blots were prepared as described previously [8].

For PAGE analysis of the samples from the “tandem IMAC/strep-tactin preparation”, one gel containing Crude/IMAC/tandem samples was subjected to SYPRO ruby staining (Invitrogen) and imaged using a Typhoon fluorescence scanning imager (GE). A second gel containing 3 × replicates of tandem samples was subjected to western blot as described previously [8]. One set of replicates was curved on the gel, complicating the densitometry boxing procedure, so it was removed from analysis, although qualitatively it presented similar results.

### Supporting data

The data set supporting the results of this article is included within the article (and its additional file).

## Endnote

^a)^As HynS is N-terminally processed, we use sequence numbers corresponding to the unprocessed HynS sequence because the exact cleavage site for the protein has not been determined experimentally.

## Abbreviations

IMAC: Immobilized metal affinity chromatography; PAGE: Polyacrylamide gel electrophoresis; SDS: Sodium dodecyl sulfate; TRC: Tryptophan/lactose hybrid promoter.

## Competing interests

The authors declare that they have no competing interests.

## Authors’ contributions

ITY conceived the experimental design, performed molecular biology, screening, and activity assays, and wrote the paper. BRC performed molecular biology, and screening assays. HOS assisted with experimental design and wrote the paper. PDW assisted with the experimental design, assisted with gels and blots, and wrote the paper. All authors read and approved the final manuscript.

## Supplementary Material

Additional file 1 **“A Broad Survey Reveals Substitution Tolerance of Residues Ligating FeS Clusters in [NiFe] Hydrogenase”. Table S1.** Doubly- and Triply- substituted mutant list. **Table S2.** Table of measured enzyme activities. **Table S3.** List of Plasmids used in this study. **Table S4.** List of Primers used in this study. **Figure S1.** Sypro-Ruby stained gel of tandem purification samples. **Figure S2.** pIY107 Sequence (genbank format). Click here for file
