# Supplementary material for: A broad survey reveals substitution tolerance of residues ligating FeS clusters in [NiFe] hydrogenase
Source: BMC Biochem. 2014 Jun 17;15:10. doi: 10.1186/1471-2091-15-10 (PMC4070099; doi:10.1186/1471-2091-15-10)
Supplement: Additional file 1 — “A Broad Survey Reveals Substitution Tolerance of Residues Ligating FeS Clusters in [NiFe] Hydrogenase”. Table S1. Doubly- and Triply- substituted mutant list. Table S2. Table of measured enzyme activities. Table S3. List of Plasmids used in this study. Table S4. List of Primers used in this study. Figure S1. Sypro-Ruby stained gel of tandem purification samples. Figure S2. pIY107 Sequence (genbank format). [file 1471-2091-15-10-S1.docx]

**Additional file 1 “A Broad Survey Reveals Substitution Tolerance of Residues Ligating FeS Clusters in [NiFe] Hydrogenase”**

**Table S1: Doubly- and Triply- substituted mutant list**

| Construct | Substitution |
| --- | --- |
| C1 | **C258D/C295D** |
| C2 | **C258D/P285D** |
| C3 | C258D/C265D/P285D |
| C4 | **C265D/C295D** |
| C5 | **C233D/C295D** |
| C6 | **C233D/C295D** |
| C7 | C233D/C258D/P285D |
| C8 | **H230D/P285D** |
| C9 | **H230D/C295D** |
| C10 | C258D/C295D |
| C11 | C258D/C295D |
| C12 | C258D/C295D |
| C13 | C258D/C295D |
| C14 | **C265D/P285D** |
| C15 | C265D/P285D |
| C16 | H230D/P285D |

**Table S2: Table of measured enzyme activities**

Screening assay values are unitless ratios to G1 evolution activity; nonzero values are geometric means and ranges in parentheses represent error-propagated geometric standard errors. Content-normalized estimates for G1 are taken by adjusting the total enzyme based on the (mature small subunit:total small subunit) ratio in the densitometric scan. For the C258D and the C295D substitutions, the content-normalized estimates are adjusted based on the western blot densitometric value relative to the G1 mature band density. “*” denotes below detection limit.

| **Substitution/Construct** | **Assay Description** | **Activity** |
| --- | --- | --- |
| G1 (H230C/P285C) | crude assay | 17.4 ± 4.8 (nmol H_2_) mg^-1^ min^-1^ |
|  | tandem-purified assay | 2.47 ± 0.28 (μmol H_2_) mg^-1^ min^-1^ |
|  | content-normalized estimate | 3.40 ± 0.49 (μmol H_2_) mg^-1^ min^-1^ |
| C78D | screening assay | 0.20 (0.15 - 0.26) |
| C78H | screening assay | --* |
| C78N | screening assay | --* |
| C78Q | screening assay | --* |
| C81D | screening assay | 0.41 (0.37 - 0.46) |
|  | crude assay | 1.69 ± 0.40 (nmol H_2_) mg^-1^ min^-1^ |
| C81H | screening assay | 0.017 (0.012 - 0.023) |
| C81N | screening assay | 0.16 (0.09 - 0.27) |
| C81Q | screening assay | 0.015 (0.008 - 0.026) |
| C158D | screening assay | 0.15 (0.12 - 0.19) |
| C158H | screening assay | 0.31 (0.24 - 0.40) |
| C158N | screening assay | --* |
| C158Q | screening assay | --* |
| C192D | screening assay | --* |
|  | crude assay | --* |
| C192H | screening assay | --* |
| C192N | screening assay | --* |
| C192Q | screening assay | --* |
| H230D | screening assay | 0.60 (0.53 - 0.68) |
|  | crude assay | 4.50 ± 0.76 (nmol H_2_) mg^-1^ min^-1^ |
| H230 | screening assay | 0.082 (0.070 - 0.096) |
| H230N | screening assay | 0.046 (0.040 - 0.051) |
| H230Q | screening assay | 0.043 (0.040 - 0.048) |
| C233D | screening assay | 0.52 (0.49 - 0.55) |
|  | crude assay | 3.66 ± 0.04 (nmol H_2_) mg^-1^ min^-1^ |
| C233H | screening assay | 0.031 (0.028 - 0.034) |
| C233N | screening assay | 0.032 (0.027 - 0.034) |
| C233Q | screening assay | 0.026 (0.025 - 0.027) |
| C258D | screening assay | 0.74 (0.67 - 0.83) |
|  | crude assay | 7.08 ± 0.81 (nmol H_2_) mg^-1^ min^-1^ |
|  | tandem-purified assay | 2.08 ± 0.15 (μmol H_2_) mg^-1^ min^-1^ |
|  | content-normalized estimate | 14.1 ± 1.9 (μmol H_2_) mg^-1^ min^-1^ |
| C258H | screening assay | --* |
| C258N | screening assay | 0.077 (0.050 - 0.12) |
| C258Q | screening assay | --* |
| C264D | screening assay | 0.58 (0.48 - 0.69) |
|  | crude assay | 3.15 ± 0.12 (nmol H_2_) mg^-1^ min^-1^ |
| C264H | screening assay | --* |
| C264N | screening assay | --* |
| C264Q | screening assay | --* |
| C273D | screening assay | 0.58 (0.52 - 0.64) |
|  | crude assay | 4.08 ± 0.09 (nmol H_2_) mg^-1^ min^-1^ |
| C273H | screening assay | 0.012 (0.009 - 0.015) |
| C273N | screening assay | 0.10 (0.091 - 0.12) |
|  | crude assay | 0.18 ± 0.08 (nmol H_2_) mg^-1^ min^-1^ |
| C273Q | screening assay | --* |
| P285D | screening assay | 0.17 (0.14 - 0.20) |
| P285H | screening assay | 0.048 (0.039 - 0.060) |
| P285N | screening assay | 0.10 (0.093 - 0.12) |
| P285Q | screening assay | 0.14 (0.11 - 0.17) |
| C292D | screening assay | 0.084 (0.074 - 0.097) |
| C292H | screening assay | --* |
| C292N | screening assay | 0.037 (0.030 - 0.046) |
| C292Q | screening assay | --* |
| C295D | screening assay | 0.48 (0.41 - 0.56) |
|  | crude assay | 7.26 ± 1.73 (nmol H_2_) mg^-1^ min^-1^ |
|  | tandem-purified assay | 2.11 ± 0.22 (μmol H_2_) mg^-1^ min^-1^ |
|  | content-normalized estimate | 7.7 ± 1.4 (μmol H_2_) mg^-1^ min^-1^ |
| C295H | screening assay | --* |
| C295N | screening assay | 0.024 (0.016 - 0.035) |
| C295Q | screening assay | 0.022 (0.014 - 0.035) |
| pIY009 | crude assay | --* |

**Table S3: List of Plasmids used in this study.**

Sequences are digitally available at: https://benchling.com/ityonemo/clusters-paper/

| **Name** | **Purpose** |
| --- | --- |
| pBC001 | Parent plasmid; derivative of pIY107, features AvrII and AgeI sites flanking hynS gene and produces dual tagged (HynS-strep, His_6_-HynL) enzyme. Note background is H230C/P285C |
| pBC002 | H230N |
| pBC003 | H230D |
| pBC004 | H230Q |
| pBC005 | H230 |
| pBC006 | C233N |
| pBC007 | C233D |
| pBC008 | C233Q |
| pBC009 | C233H |
| pBC010 | C258N |
| pBC011 | C258D |
| pBC012 | C258Q |
| pBC013 | C258H |
| pBC014 | C264N |
| pBC015 | C264D |
| pBC016 | C264Q |
| pBC017 | C264H |
| pBC018 | C273N |
| pBC019 | C273D |
| pBC020 | C273Q |
| pBC021 | C273H |
| pBC022 | P285N |
| pBC023 | P285D |
| pBC024 | P285Q |
| pBC025 | P285H |
| pBC026 | C292N |
| pBC027 | C292D |
| pBC028 | C292Q |
| pBC029 | C292H |
| pBC030 | C295N |
| pBC031 | C295D |
| pBC032 | C295Q |
| pBC033 | C295H |
| pBC034 | C192N |
| pBC035 | C192D |
| pBC036 | C192Q |
| pBC037 | C192H |
| pBC038 | C158N |
| pBC039 | C158D |
| pBC040 | C158Q |
| pBC041 | C158H |
| pBC042 | C81N |
| pBC043 | C81D |
| pBC044 | C81Q |
| pBC045 | C81H |
| pBC046 | C78N |
| pBC047 | C78D |
| pBC048 | C78Q |
| pBC049 | C78H |
| pBC050 | P285E |
| pIY009 | Plasmid featuring *Alteromonas macleodii* [NiFe] enzyme operon with the substitution HynS RR17KK driven by a single pTRC promoter. Activity should be compared to pIY003 (ref. [8]) |
| pIY107 | Parent plasmid; featuring His- and Strep- tagged *Alteromonas macleodii* [NiFe] enzyme operon in a 4x pTRC promoter system. Note background is H230C/P285C |

**Table S4: List of Primers used in this study**

Nucleotides in **RED** denote mutations introduced to effect the corresponding amino acid substitution.

| **Name** | **Sequence** | **Purpose** |
| --- | --- | --- |
| BC000AvrF | CGA AAC AAC GAT TAG CTA CAC CCT GGG GCA AGC AAT ATG GGT ATT | silently eliminates AvrII site from plasmid |
| BC001AvrR | CCT AGG TCA ATC CTC CTT AGC CGT TT | complementary primer for BC002AvrF |
| BC002AvrF | AAA CGG CTA AGG AGG ATT GAC CTA GGC ATG CGA GAG TAG GGA ACT G | creates AvrII site prior to hynS |
| IY171HynSR | CGA GTG ATA GGA TCG ACG AC | Common primer downstream of AgeI site |
| BC014DCMutF | TAT CGA CGC CCA TTT TTT GAA C | Common C233/H230 fwd |
| BC015H230QR | TGT TCA AAA AAT GGG CGT CGA TAA CAT CTG TCC TGG ATG CTT T | H230Q substitution rev |
| BC017H230R | TGT TCA AAA AAT GGG CGT CGA TAA CAT CTG TCG TGG ATG CTT T | C230H reversion rev |
| BC019H230DR | TGT TCA AAA AAT GGG CGT CGA TAA CAT CTG TCG TCG ATG CTT T | H230D substitution rev |
| BC021H230NR | TGT TCA AAA AAT GGG CGT CGA TAA CAT CTG TCG TTG ATG CTT T | H230N substitution rev |
| BC023C233NR | GTT CAA AAA ATG GGC GTC GAT AGT TTC TGT CGC AGA TGC TTT | C233N substitution rev |
| BC025C233DR | GTT CAA AAA ATG GGC GTC GAT AGT CTC TGT CGC AGA TGC TTT | C233D substitution rev |
| BC027C233QR | GTT CAA AAA ATG GGC GTC GAT ACT GTC TGT CGC AGA TGC TTT | C233Q substitution rev |
| BC029C233HR | GTT CAA AAA ATG GGC GTC GAT AGT GTC TGT CGC AGA TGC TTT | C233H substitution rev |
| BC030258NF | TTG TTC GAA CTT GGT TGT AAA GGG | Common C258 fwd |
| BC031C258NR | CCC TTT ACA ACC AAG TTC GAA CAA GTT CCA CCC ATT TTT TGC CCC CT | C258N substitution rev |
| BC033C258DR | CCC TTT ACA ACC AAG TTC GAA CAA GTC CCA CCC ATT TTT TGC CCC CT | C258D substitution rev |
| BC035C258QR | CCC TTT ACA ACC AAG TTC GAA CAA CTG CCA CCC ATT TTT TGC CCC CT | C258Q substitution rev |
| BC037C258HR | CCC TTT ACA ACC AAG TTC GAA CAA GTG CCA CCC ATT TTT TGC CCC CT | C258H substitution rev |
| BC038264F | AAA GGG CCC GAA ACC TTT AA | Common C264 fwd |
| BC039C264NR | TTA AAG GTT TCG GGC CCT TTG TTA CCA AGT TCG AAC AAA C | C264N substitution rev |
| BC041C264DR | TTA AAG GTT TCG GGC CCT TTG TCA CCA AGT TCG AAC AAA C | C264D substitution rev |
| BC043C264QR | TTA AAG GTT TCG GGC CCT TTC TGA CCA AGT TCG AAC AAA C | C264Q substitution rev |
| BC045C264HR | TTA AAG GTT TCG GGC CCT TTG TGA CCA AGT TCG AAC AAA C | C264H substitution rev |
| BC046273F | GCA ACG GTT AAA TGG AAT CAA GGC | Common C273 fwd |
| BC047C273NR | GCC TTG ATT CCA TTT AAC CGT TGC GTT TGC GTT AAA GGT TTC GGG CC | C273N substitution rev |
| BC049C273DR | GCC TTG ATT CCA TTT AAC CGT TGC GTC TGC GTT AAA GGT TTC GGG CC | C273D substitution rev |
| BC051C273QR | GCC TTG ATT CCA TTT AAC CGT TGC CTG TGC GTT AAA GGT TTC GGG CC | C273Q substitution rev |
| BC053C273HR | GCC TTG ATT CCA TTT AAC CGT TGC GTG TGC GTT AAA GGT TTC GGG CC | C273H substitution rev |
| BC052285F | ATC GAA TCT GGC CAT CCG TG | Common C285 fwd |
| BC054C285NR | CAC GGA TGG CCA GAT TCG ATG TTA AAA CTC GTG CCT TGA TTC C | P285N substitution rev |
| BC055C285DR | CAC GGA TGG CCA GAT TCG ATG TCA AAA CTC GTG CCT TGA TTC C | P285D substitution rev |
| BC057C285QR | CAC GGA TGG CCA GAT TCG ATC TGA AAA CTC GTG CCT TGA TTC C | P285Q substitution rev |
| BC059C285HR | CAC GGA TGG CCA GAT TCG ATG TGA AAA CTC GTG CCT TGA TTC C | P285H substitution rev |
| BC060292F | CTT GGT TGC TCT GAG CCC GA | Common C292 fwd |
| BC061C292NR | TCG GGC TCA GAG CAA CCA AGG TTC GGA TGG CCA GAT TCG ATA C | C292N substitution rev |
| BC063C292DR | TCG GGC TCA GAG CAA CCA AGG TCC GGA TGG CCA GAT TCG ATA C | C292D substitution rev |
| BC065C292QR | TCG GGC TCA GAG CAA CCA AGC TGC GGA TGG CCA GAT TCG ATA C | C292Q substitution rev |
| BC067C292HR | TCG GGC TCA GAG CAA CCA AGG TGC GGA TGG CCA GAT TCG ATA C | C292H substitution rev |
| BC068295F | TCT GAG CCC GAT TTC TGG GA | Common C295 fwd |
| BC069C295NR | TCC CAG AAA TCG GGC TCA GAG TTA CCA AGA CAC GGA TGG CCA G | C295N substitution rev |
| BC071C295DR | TCC CAG AAA TCG GGC TCA GAG TCA CCA AGA CAC GGA TGG CCA G | C292D substitution rev |
| BC073C295QR | TCC CAG AAA TCG GGC TCA GAC TGA CCA AGA CAC GGA TGG CCA G | C292Q substitution rev |
| BC075C295HR | TCC CAG AAA TCG GGC TCA GAG TGA CCA AGA CAC GGA TGG CCA G | C292H substitution rev |
| BC078192F | CCG CCA CTG CCT ATC GCG AT | Common C192 fwd |
| BC079C192NR | ATC GCG ATA GGC AGT GGC GGG TTG CCT GAA ATG TTT ACG ATT GGC | C192N substitution rev |
| BC081C192DR | ATC GCG ATA GGC AGT GGC GGG TCG CCT GAA ATG TTT ACG ATT GGC | C192D substitution rev |
| BC083C192QR | ATC GCG ATA GGC AGT GGC GGC TGG CCT GAA ATG TTT ACG ATT GGC | C192Q substitution rev |
| BC085C192HR | ATC GCG ATA GGC AGT GGC GGG TGG CCT GAA ATG TTT ACG ATT GGC | C192H substitution rev |
| BC086158F | GCG TCT TTT GGC GGG ATC CC | Common C185 fwd |
| BC087C158NR | GGG ATC CCG CCA AAA GAC GCG TTG GTA CCG ATG GCA ATA ATA CCC | C185N substitution rev |
| BC089C158DR | GGG ATC CCG CCA AAA GAC GCG TCG GTA CCG ATG GCA ATA ATA CCC | C185D substitution rev |
| BC091C158QR | GGG ATC CCG CCA AAA GAC GCC TGG GTA CCG ATG GCA ATA ATA CCC | C185Q substitution rev |
| BC093C158HR | GGG ATC CCG CCA AAA GAC GCG TGG GTA CCG ATG GCA ATA ATA CCC | C185H substitution rev |
| BC09481F | ACC GAA GCA ATA TTG CGC TC | Common C81 fwd |
| BC095C81NR | GAG CGC AAT ATT GCT TCG GTG TTC CCT GTG CAC TCT TGA AAG G | C81N substitution rev |
| BC097C81DR | GAG CGC AAT ATT GCT TCG GTG TCC CCT GTG CAC TCT TGA AAG G | C81D substitution rev |
| BC099C81QR | GAG CGC AAT ATT GCT TCG GTC TGC CCT GTG CAC TCT TGA AAG G | C81Q substitution rev |
| BC101C81HR | GAG CGC AAT ATT GCT TCG GTG TGC CCT GTG CAC TCT TGA AAG G | C81H substitution rev |
| BC10278F | ACA GGG TGT ACC GAA GCA AT | Common C78 fwd |
| BC103C78NR | ATT GCT TCG GTA CAC CCT GTG TTC TCT TGA AAG GGC AAC CAA ATC AC | C78N substitution rev |
| BC105C78DR | ATT GCT TCG GTA CAC CCT GTG TCC TCT TGA AAG GGC AAC CAA ATC AC | C78D substitution rev |
| BC107C78QR | ATT GCT TCG GTA CAC CCT GTC TGC TCT TGA AAG GGC AAC CAA ATC AC | C78Q substitution rev |
| BC109C78HR | ATT GCT TCG GTA CAC CCT GTG TGC TCT TGA AAG GGC AAC CAA ATC AC | C78H substitution rev |
| BC123285ER | CAC GGA TGG CCA GAT TCG ATT TCA AAA CTC GTG CCT TGA TTC C | P285E substitution rev |
| IY960PxMdF | GGG CCC GAA ACC TTT AAC GC | Proximal/Medial shuffling primer fwd |
| IY961PxMdR | GCG TTA AAG GTT TCG GGC CC | coprimer for IY960PxMdF |
| amseq30 | GGC ATT GGT TGG TCA TGC A | sequencing primer |
| amseq33rc | GCA CAC CAA CGC CAT CAT C | sequencing primer |
| amseq24 | TAG CGA AGA AGC GCA GTT AG | sequencing primer |
| amseq37rc | GAT AGG ATC GAC GAC TAA CCG | sequencing primer |

**Figure S1: Sypro-Ruby stained gel of tandem purification procedure samples. See (Yonemoto et al. 2013) for comparison to a similar gel.**

Lanes from left to right:

1. G1 crude extract
2. C258D crude extract
3. C295D crude extract
4. G1 IMAC purified
5. C258D IMAC purified
6. C295D IMAC purified
7. G1 tandem purified
8. C258D tandem purified
9. C295D tandem purified


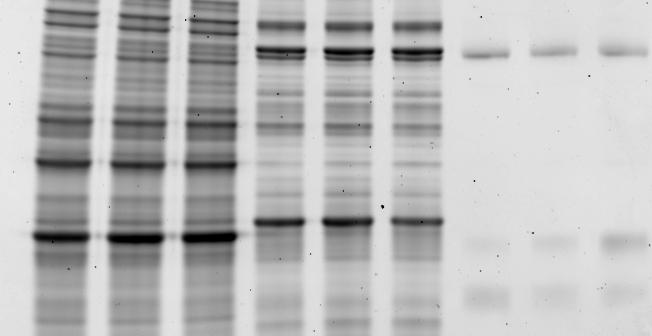


**Supplementary Figure S2: pIY107 Sequence (genbank format)**

**LOCUS pIY107 19952 bp DNA circular UNA 10-Jul-2010**

**DEFINITION**

**FEATURES Location/Qualifiers**

**rep_origin 1..589**

**/label=oriV**

**misc_feature complement(773..913)**

**/feature_type="Transfer Origin"**

**/label=oriT**

**Region 1060..1908**

**/label=NSI-up**

**CDS 2682..3622**

**/cds_type=ORF**

**/label=SpSm**

**terminator 3882..3978**

**/label=T4t**

**CDS 4302..5384**

**/cds_type=ORF**

**/label=lacI**

**promoter 5616..5678**

**/label=PTRC**

**CDS 5706..6824**

**/cds_type=ORF**

**/label=orf2**

**CDS 6839..7393**

**/cds_type=ORF**

**/label=hynD**

**CDS 7390..7824**

**/cds_type=ORF**

**/label=hupH**

**terminator complement(7825..8075)**

**/label=rrnBt**

**promoter 8076..8138**

**/label=PTRC**

**CDS 8163..9194**

**/cds_type=ORF**

**/label=hynS**

**mutation 8850..8852**

**/label=H238C**

**mutation 9015..9017**

**/label=P293C**

**CDS 9212..11113**

**/label=hynL**

**terminator complement(11166..11416)**

**/label=rrnBt**

**promoter 11417..11479**

**/label=PTRC**

**CDS 11522..11731**

**/cds_type=ORF**

**/label=hypC**

**CDS 11715..12110**

**/cds_type=ORF**

**/label=hypA**

**CDS 12100..12846**

**/cds_type=ORF**

**/label=hypB**

**terminator complement(12847..13097)**

**/label=rrnBt**

**promoter 13098..13160**

**/label=PTRC**

**CDS 13185..14318**

**/cds_type=ORF**

**/label=hypD**

**CDS 14318..16624**

**/cds_type=ORF**

**/label=hypF**

**CDS 16624..17676**

**/cds_type=ORF**

**/label=hypE**

**terminator complement(17886..18136)**

**/label=rrnBt**

**Region 18216..19228**

**/label=NSI-dn**

**ORIGIN**

**1 TTGAGATCCT TTTTTTCTGC GCGTAATCTG CTGCTTGCAA ACAAAAAAAC CACCGCTACC**

**61 AGCGGTGGTT TGTTTGCCGG ATCAAGAGCT ACCAACTCTT TTTCCGAAGG TAACTGGCTT**

**121 CAGCAGAGCG CAGATACCAA ATACTGTCCT TCTAGTGTAG CCGTAGTTAG GCCACCACTT**

**181 CAAGAACTCT GTAGCACCGC CTACATACCT CGCTCTGCTA ATCCTGTTAC CAGTGGCTGC**

**241 TGCCAGTGGC GATAAGTCGT GTCTTACCGG GTTGGACTCA AGACGATAGT TACCGGATAA**

**301 GGCGCAGCGG TCGGGCTGAA CGGGGGGTTC GTGCACACAG CCCAGCTTGG AGCGAACGAC**

**361 CTACACCGAA CTGAGATACC TACAGCGTGA GCTATGAGAA AGCGCCACGC TTCCCGAAGG**

**421 GAGAAAGGCG GACAGGTATC CGGTAAGCGG CAGGGTCGGA ACAGGAGAGC GCACGAGGGA**

**481 GCTTCCAGGG GGAAACGCCT GGTATCTTTA TAGTCCTGTC GGGTTTCGCC ACCTCTGACT**

**541 TGAGCGTCGA TTTTTGTGAT GCTCGTCAGG GGGGCGGAGC CTATGGAAAA ACGCCAGCAA**

**601 CGCGGCCTTT TTACGGTTCC TGGCCTTTTG CTGGCCTTTT GCTCACATGT TCTTTCCTGC**

**661 GTTATCCCCT GATTCTGTGG ATAACCGTAT TACCGCCTTT GAGTGAGCTG ATACCGCTCG**

**721 CCGCAGCCGA ACGACCGAGC GCAGCGAGTC AGTGAGCGAG GAAGCGGAAG AGCGCCTGAT**

**781 GCGGTATTTT CTCCTTACGC ATCTGTGCGG TATTTCACAC CGCATATGGT GCACTCTCAG**

**841 TACAATCTGC TCTGATGCCG CATAGTTAAG CCAGTATACA CTCCGCTATC GCTACGTGAC**

**901 TGGGTCATGG CTGCGCCCCG ACACCCGCCA ACACCCGCTG ACGCGCCCTG ACGGGCTTGT**

**961 CTGCTCCCGG CATCCGCTTA CAGACAAGCT GTGACCGTCT CCGGGAGCTG CATGTGTCAG**

**1021 AGGTTTTCAC CGTCATCACC GAAACGCGCG AGGCAGGATA TCCGGCAGCC GGCGGAGCGC**

**1081 TGCTTTCTTG GCAAGCGGTC GCCAGCCCCA ACGCCAGGGC TGCCAGCCCG AAACAGCGGG**

**1141 GCAAGGCAGC TTGGAAGGGC GATCGCAGCA CGGGCATGGC AATGTCTCTC TGAAGGAATG**

**1201 CAGACCTTAT TCGTACAGCC AGGGTTGAAT CGTGGGGGTC CAATCACTTA GCTCTGCTGG**

**1261 GCTAAACCAG AGAGCAATTT CCTGTTGTGC TGTTTCGATT GCATCCGAGC CATGGATGAT**

**1321 GTTGCGGCCA ATATTGACAC CAAAATCACC ACGGATGGTG CCCGGTTCTG CCGTCAGCGG**

**1381 ATTGGTAGCG CCGATCAACT TGCGAGCAGC CGCCACAACG CCTTCGCCTT CCAAGACGAT**

**1441 CGCCACGATC GGCCCAGAGG TGATGAACTC GACGAGGCCA TTGAAGAAGG GGCGCTCGCG**

**1501 GTGGACAGCA TAGTGCTGTT CGGCCAGCTC GCGACTGGGC TTCAGCTGCT TTAGGCCCAC**

**1561 CAGTTTGAAG CCTTTTTGCT CAAAGCGGCC GATGATCGTA CCGACCAAAC CCCGCTGAAC**

**1621 GCCATCGGGC TTGATGGCAA TAAATGTGCG TTCCACAGAC ATCTAGATAG TCCTCAAGAC**

**1681 GAGGCAAGCA TTGAGCTTGC CTTCCTATGG TTCGGGATCA CTGGGATTCT TGACAAGCGA**

**1741 TCGCGGTCAC ATCGCTATCT CTTAGGACTT CGCAGCGGGC GAGTCGGATT GACCCGGTAG**

**1801 GGATTTCGCC AGATCAATGC CCGTGGTTTG TTTCAGCTTC TCCAGCAAGC TAGCGATTTG**

**1861 GGTAGCGCTG CCTTCCCCTT CGCCAATCAC AGTGATCGAC TCCACGTCGA TATCTGGCAC**

**1921 GGTGCCTGAA AGCGTGACGA GCAGGGACTC GAAGCTTGCA TGCCTGCAGG TCGACTCTAG**

**1981 AGCTTTATGC TTGTAAACCG TTTTGTGAAA AAATTTTTAA AATAAAAAAG GGGACCTCTA**

**2041 GGGTCCCCAA TTAATTAGTA ATATAATCTA TTAAAGGTCA TTCAAAAGGT CATCCACCGG**

**2101 ATCAATTCCC CTGCTCGCGC AGGCTGGGTG CCAAGCTCTC GGGTAACATC AAGGCCCGAT**

**2161 CCTTGGAGCC CTTGCCCTCC CGCACGATGA TCGTGCCGTG ATCGAAATCC AGATCCTTGA**

**2221 CCCGCAGTTG CAAACCCTCA CTGATCCGCA TGCCCGTTCC ATACAGAAGC TGGGCGAACA**

**2281 AACGATGCTC GCCTTCCAGA AAACCGAGGA TGCGAACCAC TTCATCCGGG GTCAGCACCA**

**2341 CCGGCAAGCG CCGCGACGGC CGAGGTCTTC CGATCTCCTG AAGCCAGGGC AGATCCGTGC**

**2401 ACAGCACCTT GCCGTAGAAG AACAGCAAGG CCGCCAATGC CTGACGATGC GTGGAGACCG**

**2461 AAACCTTGCG CTCGTTCGCC AGCCAGGACA GAAATGCCTC GACTTCGCTG CTGCCCAAGG**

**2521 TTGCCGGGTG ACGCACACCG TGGAAACGGA TGAAGGCACG AACCCAGTGG ACATAAGCCT**

**2581 GTTCGGTTCG TAAGCTGTAA TGCAAGTAGC GTATGCGCTC ACGCAACTGG TCCAGAACCT**

**2641 TGACCGAACG CAGCGGTGGT AACGGCGCAG TGGCGGTTTT CATGGCTTGT TATGACTGTT**

**2701 TTTTTGGGGT ACAGTCTATG CCTCGGGCAT CCAAGCAGCA AGCGCGTTAC GCCGTGGGTC**

**2761 GATGTTTGAT GTTATGGAGC AGCAACGATG TTACGCAGCA GGGCAGTCGC CCTAAAACAA**

**2821 AGTTAAACAT CATGAGGGAA GCGGTGATCG CCGAAGTATC GACTCAACTA TCAGAGGTAG**

**2881 TTGGCGTCAT CGAGCGCCAT CTCGAACCGA CGTTGCTGGC CGTACATTTG TACGGCTCCG**

**2941 CAGTGGATGG CGGCCTGAAG CCACACAGTG ATATTGATTT GCTGGTTACG GTGACCGTAA**

**3001 GGCTTGATGA AACAACGCGG CGAGCTTTGA TCAACGACTT TTGGAAACTT CGGCTTCCCC**

**3061 TGGAGAGAGC GAGATTCTCC GCGCTGTAGA AGTCACCATT GTTGTGCACG ACGACATCAT**

**3121 TCCGTGGCGT TATCCAGCTA AGCGCGAACT GCAATTTGGA GAATGGCAGC GCAATGACAT**

**3181 TCTTGCAGGT ATCTTCGAGC CAGCCACGAT CGACATTGAT CTGGCTATCT TGCTGACAAA**

**3241 AGCAAGAGAA CATAGCGTTG CCTTGGTAGG TCCAGCGGCG GAGGAACTCT TTGATCCGGT**

**3301 TCCTGAACAG GATCTATTTG AGGCGCTAAA TGAAACCTTA ACGCTATGGA ACTCGCCGCC**

**3361 CGACTGGGCT GGCGATGAGC GAAATGTAGT GCTTACGTTG TCCCGCATTT GGTACAGCGC**

**3421 AGTAACCGGC AAAATCGCGC CGAAGGATGT CGCTGCCGAC TGGGCAATGG AGCGCCTGCC**

**3481 GGCCCAGTAT CAGCCCGTCA TACTTGAAGC TAGACAGGCT TATCTTGGAC AAGAAGAAGA**

**3541 TCGCTTGGCC TCGCGCGCAG ATCAGTTGGA AGAATTTGTC CACTACGTGA AAGGCGAGAT**

**3601 CACCAAGGTA GTCGGCAAAT AATGTCTAAC AATTCGTTCA AGCCGACGCC GCTTCGCGGC**

**3661 GCGGCTTAAC TCAAGCGTTA GATGCACTAA GCACATAATT GCTCACAGCC AAACTATCAG**

**3721 GTCAAGTCTG CTTTTATTAT TTTTAAGCGT GCATAATAAG CCCTACACAA ATTGGGAGAT**

**3781 ATATCATGAA AGGCTGGCTT TTTCTTGTTA TCGCAATAGT TGGCGAAGTA ATCGCAACAT**

**3841 CCGCATTAAA ATCTAGCGAG GGCTTTACTA AGCTGATCCG GTGGATGACC TTTTGAATGA**

**3901 CCTTTAATAG ATTATATTAC TAATTAATTG GGGACCCTAG AGGTCCCCTT TTTTATTTTA**

**3961 AAAATTTTTT CACAAAACGG TTTACAAGCA TACTAGAGGA TCGGCGGCCG CGGATCTGGG**

**4021 CCGCGGGTCA TGGCTGCGCC CCGACACCCG CCAACACCCG CTGACGCGCC CTGACGGGCT**

**4081 TGTCTGCTCC CGGCATCCGC TTACAGACAA GCTGTGACCG TCTCCGGGAG CTGCATGTGT**

**4141 CAGAGGTTTT CACCGTCATC ACCGAAACGC GCGAGGCAGC AGATCAATTC GCGCGCGAAG**

**4201 GCGAAGCGGC ATGCATTTAC GTTGACACCA TCGAATGGTG CAAAACCTTT CGCGGTATGG**

**4261 CATGATAGCG CCCGGAAGAG AGTCAATTCA GGGTGGTGAA TGTGAAACCA GTAACGTTAT**

**4321 ACGATGTCGC AGAGTATGCC GGTGTCTCTT ATCAGACCGT TTCCCGCGTG GTGAACCAGG**

**4381 CCAGCCACGT TTCTGCGAAA ACGCGGGAAA AAGTGGAAGC GGCGATGGCG GAGCTGAATT**

**4441 ACATTCCCAA CCGCGTGGCA CAACAACTGG CGGGCAAACA GTCGTTGCTG ATTGGCGTTG**

**4501 CCACCTCCAG TCTGGCCCTG CACGCGCCGT CGCAAATTGT CGCGGCGATT AAATCTCGCG**

**4561 CCGATCAACT GGGTGCCAGC GTGGTGGTGT CGATGGTAGA ACGAAGCGGC GTCGAAGCCT**

**4621 GTAAAGCGGC GGTGCACAAT CTTCTCGCGC AACGCGTCAG TGGGCTGATC ATTAACTATC**

**4681 CGCTGGATGA CCAGGATGCC ATTGCTGTGG AAGCTGCCTG CACTAATGTT CCGGCGTTAT**

**4741 TTCTTGATGT CTCTGACCAG ACACCCATCA ACAGTATTAT TTTCTCCCAT GAAGACGGTA**

**4801 CGCGACTGGG CGTGGAGCAT CTGGTCGCAT TGGGTCACCA GCAAATCGCG CTGTTAGCGG**

**4861 GCCCATTAAG TTCTGTCTCG GCGCGTCTGC GTCTGGCTGG CTGGCATAAA TATCTCACTC**

**4921 GCAATCAAAT TCAGCCGATA GCGGAACGGG AAGGCGACTG GAGTGCCATG TCCGGTTTTC**

**4981 AACAAACCAT GCAAATGCTG AATGAGGGCA TCGTTCCCAC TGCGATGCTG GTTGCCAACG**

**5041 ATCAGATGGC GCTGGGCGCA ATGCGCGCCA TTACCGAGTC CGGGCTGCGC GTTGGTGCGG**

**5101 ATATCTCGGT AGTGGGATAC GACGATACCG AAGACAGCTC ATGTTATATC CCGCCGTTAA**

**5161 CCACCATCAA ACAGGATTTT CGCCTGCTGG GGCAAACCAG CGTGGACCGC TTGCTGCAAC**

**5221 TCTCTCAGGG CCAGGCGGTG AAGGGCAATC AGCTGTTGCC CGTCTCACTG GTGAAAAGAA**

**5281 AAACCACCCT GGCGCCCAAT ACGCAAACCG CCTCTCCCCG CGCGTTGGCC GATTCATTAA**

**5341 TGCAGCTGGC ACGACAGGTT TCCCGACTGG AAAGCGGGCA GTGAGCGCAA CGCAATTAAT**

**5401 GTAAGTTAGC GCGAATTGAT CTGGTTTGAC AGCTTATCAT CGACTGCACG GTGCACCAAT**

**5461 GCTTCTGGCG TCAGGCAGCC ATCGGAAGCT GTGGTATGGC TGTGCAGGTC GTAAATCACT**

**5521 GCATAATTCG TGTCGCTCAA GGCGCACTCC CGTTCTGGAT AATGTTTTTT GCGCCGACAT**

**5581 CATAACGGTT CTGGCAAATA TTCTGAAATG AGCTGTTGAC AATTAATCAT CCGGCTCGTA**

**5641 TAATGTGTGG AATTGTGAGC GGATAACAAT TTCACACAGG AAACAGACCA TGGAggagga**

**5701 actagATGGC TTCCATTATT GGTAGCGGCT TTTTGATCAT TGCACCACTG CTGCATTCGG**

**5761 TAATGGGGAA ATGGGCTCTG CTTGGAATAG TGTTGTTGTC CATTCTGGCA TATGCGCTGG**

**5821 GGTCGGTTAT TCGCTTCAAT ATACGCCATG CAGAACCTGA GCTAGCTAGC AATCAACAAA**

**5881 GCAGTATTGT GACGCTTGAA AAAGCAAGTC AATGGGCGCT TGGTGCGGCA TATGCCATTT**

**5941 CCGTGGCGTT TTATATTAGT TTGTTTGCAG CGTTTGTTTT TGATCGTTTA TCAATCTCTG**

**6001 ATACGACGTA CATCAAACTG TTTACCAGTG GGTTGCTTGT CATCATTACG GTGGTCGCTT**

**6061 GGCTCCGTGG TGCTAGGGGG CTAGAGACCA TTGAGCTATT CGCCGTGACA ATAAAGTTGG**

**6121 CTATTATAGT AGGTGTGCTC GCAGCGCTGG CAACCTACGA CATACAAGTA CAAAGTGCTT**

**6181 GGTTTCAACA TGAAGCGATA CAGGCATTGA GCCATTTTGA AACTGTTAGC ATGCTCGCAG**

**6241 GCATGCTCAT GGTAACGCAA GGGTTCGAGA CAACCCGTTT TATGGGGAAT AACTACACGC**

**6301 CAGAACAGCG CATTAAAGCG AGTCGTTATG CTCAGTGGAT CGCTATTTTT CTCTATGTTG**

**6361 TTTTTATCGG TCTAACGTGT CCTATTTTTC TGGATTTTCC GATCACGGAA TTAAACGAAA**

**6421 CAACGATTAG CTACACCCTA GGGCAAGCAA TATGGGTATT GCCGATTTTA TTACTGGTTG**

**6481 CCGCCACGGC AAGTCAATTA AGTGCAGCAT TAGCCGATAC TATCGGTGGC GGAGGCCTCT**

**6541 TAAAAGAGCT TTTCCATTTA CCTATCTCCC CGCAATTTTA CTACGTTTTA GTCATCGCCA**

**6601 TCGCAGGTAT ATTAGTTTGG TCATCCAACG TATTTGAAAT TATTAATCTC GCTTCAAAAG**

**6661 GGTTTGCACT TTATTACCTG ATCCAAACAA TAATCGCGGT CAAACTCGTG ATGCGTCAAA**

**6721 GCACAGGTCA ACGCATTAGG TCAACAAAAT TACTTGGTCT ATCGGTGATC GTTCTCTGTT**

**6781 TATTCTTTGT GATTGGCTGG TCAATTCCAG CACCTCATAG CTAATAAGGA AAATACCGAT**

**6841 GCTATCAGTA GAAACTACAC CACCAATTGA CCAAATCAAA AACGCGGCTA AGCGCTACGC**

**6901 TATCATTGGT GTTGGAAATC TATTGCAGCG AGATGATGGC GTTGGTGTGC ACGCGGTTCG**

**6961 TGCTATGCAA CCCCTGCTAA AGTCTTATTC AAATGTATCT TGCATTGACG CTGGGACACT**

**7021 GAGCTATGAA CTTTTGGAGT GGGTTGCGAG TTCCGATCAT ACGATAGTCA TCGACGCGGC**

**7081 TTATATGCGC TGTCCGCCTG GCACGGTGAG AGTGTTTGAG AACGAGGCAA TTGCGGATCA**

**7141 GTTTCAGCAA GCGACGACCC ATTCCGTTCA CCAGATCACG TTACGCGACA CATTGTTGAC**

**7201 CAGCCAAACG TTATATTCAA AACCTTCTGC GGTGACGTTA ATTGGCGTAG AGCCGGAGGC**

**7261 GATTGAGTGG GGAACCTCTT TGAGTCCAAA GGTAAATGCA GCGCTATCAT CGATCATGGA**

**7321 TATCGCATTT GCTTGCTTAT CAGGCGACAA CGTTCAGACC TATAAACCGG CAACCCAAAA**

**7381 GGAGGCACGA TGACCGAAGA ACTCATTTTT ATGACATCAA ATGCTGATAA AACGGGCAAC**

**7441 GTAATGCCGC TTTTGCATCA GATACGTCAT GCGTTGTCTC AGTTAATTGA ACGACAAGAA**

**7501 CAAACCACGA TTGATCTGCG ACGATTGCCT TTAAGCGCTA GCGAAGAAGC GCAGTTAGAA**

**7561 GCTTTTCTTG GGCATGGAGA GGTTAAAGCT GATATTCAGG CGCTGGGTGA TACGGTGCTA**

**7621 ATCGAATCAC GCTACGCAGG CGTTTGGCTT GAAATTCACT ATAACGAAGA TGTGGAGATC**

**7681 ATGGGCAAAT ACGTTCATAT CTGTACCTGT CCACCAATCA TAAAATCTCA GCCAGAAGAT**

**7741 ATGGTGTTAT CGCTCAGCAA TATAGTGTCT GACATCCATT CGTTGTCTCA TCAATCTTCT**

**7801 GATGAAACGG CTAAGGAGGA TTGACATGCG AGAGTAGGGA ACTGCCAGGC ATCAAATAAA**

**7861 ACGAAAGGCT CAGTCGAAAG ACTGGGCCTT TCGTTTTATC TGTTGTTTGT CGGTGAACGC**

**7921 TCTCCTGAGT AGGACAAATC CGCCGGGAGC GGATTTGAAC GTTGCGAAGC AACGGCCCGG**

**7981 AGGGTGGCGG GCAGGACGCC CGCCATAAAC TGCCAGGCAT CAAATTAAGC AGAAGGCCAT**

**8041 CCTGACGGAT GGCCTTTTTG CGTTTCTACT ACTAGTTGAC AATTAATCAT CCGGCTCGTA**

**8101 TAATGTGTGG AATTGTGAGC GGATAACAAT TTCACACATA CTAGAAAGAG GAGAAATACT**

**8161 AGATGGCGCT ACCAACATTA AACAAGCAGT TACAAGCGTC CGGTATCTCA AGACGCACAT**

**8221 TTCTTAAGTT TTGCGCTACA ACGGCGTCTT TACTGGCATT GCCGCAAAGC GCTGTTGCCG**

**8281 ATTTGGCGAC TGCCCTTGGC AATGCGAGAA GACCCTCTGT GATTTGGTTG CCCTTTCAAG**

**8341 AGTGCACAGG GTGTACCGAA GCAATATTGC GCTCTCATGC TCCCACATTG GAAAGCCTTA**

**8401 TTTTCGATCA TATTTCGTTG GATTATCAGC ATACGATAAT GGCTGCTGCG GGAGAGCAAG**

**8461 CTGAAGACGC TAGGCGTGCG GCGATGAACG CGCACAAAGG GCAATATTTG TTGTTGGTTG**

**8521 ATGGTTCGGT TCCGGTGGGT AACCCAGGAT ACTCAACGAT CAGTGGCATG AGTAATGTCG**

**8581 ATATGCTGAG AGAATCGGCA AAAGATGCTG CGGGTATTAT TGCCATCGGT ACCTGCGCGT**

**8641 CTTTTGGCGG GATCCCTAAA GCAAACCCAA ATCCGACGGG GGCAGTGGCA GTAAGCGACA**

**8701 TTATTACAGA CAAGCCAATC GTAAACATTT CAGGCTGTCC GCCACTGCCT ATCGCGATTA**

**8761 CAGCTGTGTT GGTTCATTAC CTGACGTTTA AGCGTTTCCC TGATCTCGAC GAATTACAAC**

**8821 GCCCACTCGC TTTTTTTGGT GAAAGCATCT GTGACAGATG TTATCGACGC CCATTTTTTG**

**8881 AACAACGTAA ATTTGCAAAA TCGTTTGATG ATGAGGGGGC AAAAAATGGG TGGTGTTTGT**

**8941 TCGAACTTGG TTGTAAAGGG CCCGAAACCT TTAACGCATG TGCAACGGTT AAATGGAATC**

**9001 AAGGCACGAG TTTTTGTATC GAATCTGGCC ATCCGTGTCT TGGTTGCTCT GAGCCCGATT**

**9061 TCTGGGATAA AAGCAGCTTT TACCAAGCCT TGGGTCCATG GGAGTGGTAC AAATCCAAAC**

**9121 CCGGCAAAGG TGCACAGAAG CATGCTGGGA AAAACTCACG TAGCGCTTGG TCCCACCCGC**

**9181 AGTTCGAAAA ATAAGATAAT AGGCAAGGCT CATGGCACAT CACCACCACC ATCACGCAGA**

**9241 AAATACAGCA AGTAACAACC GGTTAGTCGT CGATCCTATC ACTCGAATTG AAGGGCATCT**

**9301 TCGAATAGAA GCTGAAATGG ATGGGAATAC CATCAAACAG GCGTTCTCAT CAGGCACGTC**

**9361 TGTTCGGGGA ATTGAACTGA TTTTACAAGG CAGAGATCCG CGTGACGCTT GGGCTTTTGC**

**9421 GCAACGTATC TGTGGCGTCT GCACGCTGGT GCATGGTATG GCATCGGTGC GTGCTGTCGA**

**9481 AGATGCAATT AGAAAAGCTT GGCGGTCAAA CGCAAAATTA GGGGTTGCCA TTGGAAAGCC**

**9541 CTCCATGACA TCTATGCCAA AAGGACCGAT GCAACATGGT AAAAAAGGGC ACCGACAGTC**

**9601 ACGTACTTCA ATAGGCGTAC TGAGTGAAGC AGAAATGGCT ATCCCTCAAA ATGCACAACT**

**9661 GATAAGAAAC ATCATGATTG CAACCCAATA TGTGCATGAT CACGTGATGC ATTTTTATCA**

**9721 CTTGCATGCC CTAGATTGGG TTGACGTTGT TTCTGCACTA GATGCAGATC CAACGAGAAC**

**9781 CGCTACGCTC GCCGGTCAAT TGAGTGATTA TCCTCGTTCA TCGCCGGGAT ATTTCAAAGA**

**9841 TGTGAAGCAA AAAGTCAAAA CGCTGGTTGA GTCTGGGCAG CTAGGGATAT TCAGTAACGC**

**9901 GTATTGGGGG CATCCTGGCT ATAAACTGCC ACCTGAAGTT AACTTGATGG CATTAGCACA**

**9961 TTATTTAGAT GCGCTAACGT GGCAGCGTGA AGTTGTAAAA GTTCACACCA TATTTGGAGG**

**10021 GAAAAACCCT CATCCTAATT TTGTCGTTGG CGGCGTGCCT TCACCGATTA ATCTCAATGC**

**10081 GTCAACGGGT ATTAACACGA GTCGATTAGT GCAACTACAA GATGCTATCA CGCAAATGAA**

**10141 GAGCTTTGTC GATCAGGTGT ATTACCCCGA TATTGTGGCG ATTGCGGGTT ATTACAAAGA**

**10201 GTGGGGGACA CGAGGGGAAG GGCTGGGTAA CTTTCTTACC TATGGAGACT TACCTATGAC**

**10261 ATCAATGGAT GACCCTGATT CTTTCTTGTT TCCACGAGGT GCAATACTTG GTCGAGACTT**

**10321 GAGTAAAGTG CATGACCTTG ATCTAGATGA TCCCTCTGAA ATTCAAGAAT TCGTCTCTTC**

**10381 CTCCTGGTAT CGATATAGTG GAGGGAACGC AAGTGGTTTA CACCCTTTTA ATGGACAAAC**

**10441 AACACTCGAG TATACTGGTC CGAAACCGCC TTACAAGCAC CTAAATGTAG GGGCTGAATA**

**10501 TTCATGGTTG AAAAGTCCGC GTTGGAAAGG CCATGCGATG GAGGTGGGAC CGCTGGCTCG**

**10561 CGTGCTAATG ATGTATGCTA AAAAAGATGC CGCTGCGCAA GACATCGTTA ATCGATCTCT**

**10621 TTCTATCTTG GATTTAGAGA CCTCTGCACT TTTCTCCACA CTCGGTAGGA CGCTCGCCAG**

**10681 AGCAGTGGAA ACAAAAATTG TGGTTAACCA GCTACAGTCT TGGTATGACC AACTATTGGA**

**10741 TAATATCGCA AAGGGCGATA CCGATACGTT TAACCCTCTG TATTTTGACC CTACCAATTG**

**10801 GCCAATTAAA GGCCAGGGGG TAGGCGTGAT GGAAGCGCCT CGTGGCGCGT TGGGGCATTG**

**10861 GTTGGTCATG CAAAATGGCA AAATTGAGAA TTACCAATGT GTCGTGCCTA CGACATGGAA**

**10921 CGCTGGACCT CGAGATCCCA ACTCACAGGC AGGTGCTTAT GAAGCCGCTC TGCAAGATAA**

**10981 ACATACGCTA CATGATCCTG ACCAACCTTT AGAGATTTTG CGAACACTTC ATAGTTTTGA**

**11041 CCCCTGCTTA GCATGTGCCG TGCACGTAAT GGACGAAACA GGGGAAGAGC GTTTGCGTCT**

**11101 AAAAGTTCGT TAAgatcctc tacgccggac gcatcgtggc cggTCGAGCT CAAGGAGGAA**

**11161 TAACACATGC GAGAGTAGGG AACTGCCAGG CATCAAATAA AACGAAAGGC TCAGTCGAAA**

**11221 GACTGGGCCT TTCGTTTTAT CTGTTGTTTG TCGGTGAACG CTCTCCTGAG TAGGACAAAT**

**11281 CCGCCGGGAG CGGATTTGAA CGTTGCGAAG CAACGGCCCG GAGGGTGGCG GGCAGGACGC**

**11341 CCGCCATAAA CTGCCAGGCA TCAAATTAAG CAGAAGGCCA TCCTGACGGA TGGCCTTTTT**

**11401 GCGTTTCTAC TACTAGTTGA CAATTAATCA TCCGGCTCGT ATAATGTGTG GAATTGTGAG**

**11461 CGGATAACAA TTTCACACAT ACTAGAAAGA GGAGAAATAC TAGATGTGTC TCGCTATCCC**

**11521 CATGAAAGTC ATTGCAATTA AAGGCTTTAA CGCAACGTGT GAAGCGAAAG GTGTCTCTCG**

**11581 CGAGGTGAGT TTACATTTAG TGCAAGGGCT AGAGGTCAAA GTTGGCGACT ATGTGATGGT**

**11641 ACATGTTGGT TATGCTCTCC AAGTGATCAC CTATGATGAA GCGCAGGTGA CATGGGAGAT**

**11701 GCTCGATCAG GTCATTGCTT ACGATGCATG AAATTAGTCT TTGCTATAGC TTGCTTGACA**

**11761 CAGTCGCTGT TCACCAGCGA GCAAACATGA ATAAGTCTGT CAGTCTGGTT CACGTCAAAG**

**11821 TCGGCCCACT GTCAGGTGTT GAGCCTGACT TGCTGCACCA TGCGTTCTTG GCGTGTAGAA**

**11881 CTCACACCAT TGGCGATCAG GCAACGCTTA GAATTGACAC AAGCGCTATC AAGATACGCT**

**11941 GTAAATTGTG TGGCGAATTC AGCCGAGTTT CGGTGAATAG CATCCTATGT GCAAGTTGCG**

**12001 GTGCTTGGCA AACCGACCTC ATTGAGGGCG ATGAATTTAT ATTGCAACGA ATAGAGTTTA**

**12061 TCGCACCTGA CAATTTTACA GAATCAAAGG AGAGAAAACA TGTGCGGTAA CTGTGGTTGC**

**12121 GAAACGCAAG TAAAATCAGA GAAAACTGAC AAGCCATCAG CAAATATCGT TGAGGTTCAC**

**12181 GCAAATTTAA AAAGCAACAA TGACAACCAA GCGATAGCAA ACCGTGCTTT ATTTGACGCA**

**12241 CATAATGTAC TAGTGATTAA TTTGATGTCA TCCCCAGGCA GTGGAAAAAC CAGGCTACTC**

**12301 GAAGAAACGA TACGTGCGCT TAAGTCGCAA TACGCGATGG CGGTGATTGA AGGCGACCTC**

**12361 GAAACTGAAA ATGACGCAAA TAGAATTAGA CGTCATGGTG TGCAGGCGGA GCAAATCGCC**

**12421 ACGGGCCAAG GGTGTCACTT GGACGCCAGT ATGGTGCAAA AGGTACTTGG AAGATTTAGC**

**12481 TTACAAAAAC TAGACGTTTT GTTTATAGAG AATGTTGGCA ATTTGATTTG TCCAGCGTGT**

**12541 TTTGATTTAG GACAGCACTT AAACATTATT TTGCTGTCCG TACCAGAAGG TGATGATAAG**

**12601 CCTGAAAAGT ACCCAGTGAT GTTTCGTGCC GCGGACGTCA TGCTTATTTC TAAGACCGAT**

**12661 TACTTGCAAT TTCACGATGA ATTCAATGTA CCTCGCGCGA TTGAATCATT TCGTAAAGTG**

**12721 GGCAACGATG CGCCCGTTCT CGAAGTGTCT TCATTGAAAA GTCAACATCT AGAGCGTTGG**

**12781 TTTTCTTATT TTACGCACGC CATTACAGCT CATCAGTCTC AGTCGTTACA AAATAGCGGA**

**12841 GTCTAACATG CGAGAGTAGG GAACTGCCAG GCATCAAATA AAACGAAAGG CTCAGTCGAA**

**12901 AGACTGGGCC TTTCGTTTTA TCTGTTGTTT GTCGGTGAAC GCTCTCCTGA GTAGGACAAA**

**12961 TCCGCCGGGA GCGGATTTGA ACGTTGCGAA GCAACGGCCC GGAGGGTGGC GGGCAGGACG**

**13021 CCCGCCATAA ACTGCCAGGC ATCAAATTAA GCAGAAGGCC ATCCTGACGG ATGGCCTTTT**

**13081 TGCGTTTCTA CTACTAGTTG ACAATTAATC ATCCGGCTCG TATAATGTGT GGAATTGTGA**

**13141 GCGGATAACA ATTTCACACA TACTAGAAAG AGGAGAAATA CTAGATGTAC AGTGTTGAAT**

**13201 CGTTGCTAAG AGACATTAAG AATACCTCGC TTTCAAAACC GTTTCGTATT CTTAATGTTT**

**13261 GCGGTGGCCA TGAGCGTGCG ATTACGCGCG CTGGATTTAG AACGTTGTTT CAGCACAATA**

**13321 TTCATCTGAT ACCTGGGCCA GGGTGCCCCG TTTGTATTTG CCCCGAAGAG GATATTGCAT**

**13381 ACGCGATACA CTTAGCCATT AACGAAAATG TCGTTATCGT TAGTTTTGGT GACATGCTCC**

**13441 GCGTCCCAGT CGAACACGAA ATGGGTGGCT GTAGCTCATT GATAGACGCA AAAAATCAAG**

**13501 GGGCCGATAT CCGTCCGATA TCTTCTCCTC AAGAAGCGGT ATCTATAGCA TTGGAAGAAC**

**13561 CTCGTAAGGT TATTGTGTTT TTTGCTGTTG GTTTTGAAAC GACTATGGCA CCGATTGCAG**

**13621 CAACACTGCT TAATGATTTA CCCAATAACT TTAAAGTATT ATTGTCTGGA CGACTTACTT**

**13681 GGCCAGCAGT TGCCCATGTG CTTGAGAGTC AACCGAATAC TTTTGATGCA CTGATTGCGC**

**13741 CAGGCCACGT TGCGACAATT ATGGGTAGTG AAGAATGGCA GTTTGCAATC GACCATCATA**

**13801 ATTTGCCTGT CAGTATTGCT GGTTTTCATC CAGAAAGTTT GCTGTTGTCG CTCCAAACGC**

**13861 TGCTCGGTAA TTGTTCAAAT AAAGTCGTCA CACTGAGTAA CCGCTATCCC GAAGTGGTAA**

**13921 AGCAAAACGG CAATGCAGCA GCTAAAGCAA TAATCAATAA AGCGTTCACT ATCGTTGACG**

**13981 CACATTGGCG AGGAATTGGT GTTATTCCTG GTTCTGGATT TTCCTTTGCT TCGAGACTAT**

**14041 CGCACCTAGA CGCCACAAAC GATTATGCGC CAGTCGATTT CCCTTCGCAA TGTGCGCAAA**

**14101 ACGTTCCTGA AACCACATCG CCCTGTGAAA AAGTGATCCT CGGAAAAATG GCACCTGATG**

**14161 CGTGCCCGTT TTTTGGTCAG GAATGCAAAC CAGCATCCCC TAAAGGCGCA TGTATGGTGT**

**14221 CTGATGAGGG GGCTTGCCGA ATTTGGTATA GCTCTGGTGA GCGCTCGATA ACAAATGTTA**

**14281 TCAAAAAGGG GAATACGCTA AAGGTGGAGA TGAAGTGATG ACAACTGCCC GCACATATGT**

**14341 TGCCAATACG AATAAGAGTA AAACTATCGC CTATGAGTTG AACATTACTG GAGTCATCCA**

**14401 GGGGGTTGGG TTCAGACCGT TAGTTTACTG CTTAGCGTAT GAAAAAAGTA TTGTTGGTTG**

**14461 GGTACAAAAC GACTGTGGAT GTGTGCGAAT ACACGCTGAA GGCACTGAGC TAGACGTTGA**

**14521 ACAATTTGTA TTCGACTTAC TGAACAATGG GTCGCTAGTT TCCATATCCT TGCTGGAAAA**

**14581 AAAGCAGGTT AAACTGAGTA ATGTAGACTC GTTCACGATA AAAGAAAGCG GTCATGATAT**

**14641 AGCGTGCGGG GCGATATCAA TCCCCAAAGA TGTCTATTTG TGTGGAGCGT GTAAAGCTGA**

**14701 GTTACTGTCA AATACTAACC GACGTGGCAC CTATAGCTTT ATCGCTTGCA GTAAATGTGG**

**14761 TCCACGATTT TCTATGCTGC GAGCAATGCC ATACGACAGA AAGAACATGA GTATGTCAGC**

**14821 ATTTCCAATG TGTGAGTTAT GTCGTAAAGA ATATCAATCG CCAACTGATA GGCGCTTTCA**

**14881 TGCTCAACCA ATAAGCTGTC GCGTATGCGG GCCTGAAGTA TTTTGTTCAA CTGTCGGCGG**

**14941 GCGCGTGGTC TCGCAAGGCG ATGTTGATGT TGTAGCAACG GTTGTCGCGT ATTTGAATCA**

**15001 AGGCAAAATT GTCGCGTTAA AAAGTATAGG CGGTTATCAC TTGCTCTGTG ACGCACAAAG**

**15061 CACTGAAGCG GTAGACCTTT TACGCCAGCG TAAAAATAGA CCAGATAAAC CTTTCGCGGT**

**15121 CATGCTCCCA GAGCCTCGTG CAGACGAAAC AAGTGAGGAT TGGTTAGATA AATGCGTTGC**

**15181 TGTAGACAGT CATCAAAGAG CGCTCTTACT AAGCCCCGTG CGACCCATTT TACTTGCAAA**

**15241 AAAGCAATCG AATGTACCCA TTGCAGATAA CGTAGCGCCC ATGCTTCGTG ATTTGGGGGT**

**15301 GATGTTGCCG TGCAGCGGAC TGCATTTACT CATTATGCAG CAATTCAATC GCCCCATGGT**

**15361 GGCTACGTCA GGAAATATTT ACGGTGAACC GATGGTCACA TCGACGTCAT CGGCACAGCA**

**15421 ACACCTTTCG ATGTTGGCAG ACATCTTCGT GCACCATAAT CGAGACATTT TTCACAAACT**

**15481 GGATGATTCT GTGCTGCGAA CGCTTAAGTC AGGCACCATT CCAATTCGCC TGGGGCGGGG**

**15541 TATAAGTCCG ATAGAATTAT CATTGCCATA TACTGTGCAG GAACCAATGC TTGCTGTTGG**

**15601 CGCGAATCAG AAAAATACAG TCGCAATAGC TTTGAACAAT CGACTTATCG TAACACCGCA**

**15661 TATTGGCGAC CTCGATTCTC CGATAATGCA AGCGCACTTT GAAAAATGTA TCAGTGACTT**

**15721 TCAAGCTCTC TATCATGTTC ATCCACAGCG TATTCTTTGC GATAGCCATC CTGGCTATAT**

**15781 CAGTTCCCGT TGGGCCAAAG GCAGTGAACT ACCGACTACT AAAGTTTTAC ATCACCACGC**

**15841 TCATGCATCA GCGTGGGCAT TAGCTGCTGA AATATCGACA CCAAGCTTGG TTTTCGTGTG**

**15901 GGATGGAACG GGATTAGGCG AGAATGGCGA GATGTGGGGA GGGGAAGTTT TTTGTGGGAC**

**15961 GCCGGGCAAT TGGAAACGTG TTGCCTCATT AGCGCCTATG AAGTTGCAAG GCGGCAACAA**

**16021 GGTTCCAAAT CAACCATGGC GAAGTGCAGC ATCACTAGTG TGGGGTACTA ATTTCACCGG**

**16081 CATTGATTTT AAACCATTTG ATCCACAACA ATTAAGCTAC AACGCATGGC AAAACGAACT**

**16141 CAATTGTCAT GTTAGTAGTT CAATGGGAAG ACTGTTCGAC GCGGCGGCAT TTGCCTGCTG**

**16201 TGAACAGAGC GAAGTCTCTT ATGAAGGGCA GGCGCCGATG ATGTTGGAAA GCGTTGCGTC**

**16261 GACGCCCTCT AAGGTTATGC CGTTAACGTT GGTCCAGAAA AACGACGAAT TTATTCAAAT**

**16321 GGATTGGCGA ACACTGGTAC CGGAACTATT TGATTTCAAA GTGTCTCCTC AAACTCGTGC**

**16381 TGCAAATTTC CACGCTTCGT TAATTGAAAC GGTATGGGAG GTAACCCTAC ATTGCCGTAA**

**16441 AAAGTTTAAT TTTGACCACA TTGGATTGGC AGGAGGCGTC TTCCAAAATC GCATTTTGTG**

**16501 TGACGGTATA TCACAACGGT TTTTACAATC AGACATTCCT GTGGTTATTC CAAGTAGTTT**

**16561 GCCATTAAAC GATGCCGCAA TAAGTGTTGG GCAAATCCAC GAATACTGTT CACGGAGGTC**

**16621 ATAATGAAAA GCGCAGAGGG CAATAATCAT TCATCATCAG AGCCGTTGGG CACGGAATCT**

**16681 ATTCAATTAG CTCAGGGGAA CGGCGGAACA CTGACAAAAA GACTCATTGA TCATGTTTTT**

**16741 AAAAATAAAG CGAACAAAGA TCTAGATCTG CTTCATGATG CCGCCACAGT AACTTTTGAT**

**16801 GGTCCTTATC TGAGTATAAC AACGGATAGC TTCGTTGTAT CCCCCCCAAC ATTTCCAGGC**

**16861 GGCAATGTAG GCGCGTTGAG TGTTTACGGT ACCGTCAATG ATTTAGCAGT CGTTGGGGCA**

**16921 ACACCACGGT ACATCAGTAC TGCATTTATT ATTGAAGAAG GCTTTTCCCT TTCAACATTG**

**16981 AAACAAATCG TTAATGGAAT GCATCAAGCT GCCGAAGAAA CGAAGGTCGC CATTGTAACA**

**17041 GGTGATACGA AAGTTGTCCC GAAAGGCAGT GGTGGCGGCG TTTTTATCAA TACAACGGGT**

**17101 GTTGGAGACA GCCGAGATGC GCCGATTTGG GATACCAGTC TTATAAAGGC AGGCGATCAT**

**17161 GTACTGGTTA GTGGTTCTGT AGGTGATCAC GGTGCGTGTG TCCTGCTTGC TCGCGAGGAC**

**17221 TATGGTTTGC AAGGTCAGCT AAAGTCTGAT TGTGGAAGCG TTGTTCCTCT TATTGATCCA**

**17281 ATCAAGCATT TGGAAGGTGT TCGTTTTGTG CGTGACCCAA CTCGTGGAGG GTTGTCAGTA**

**17341 TTACTCCATG ACGTTGCTAA TGAAACAGGC TTCGATATCG AGTTAATTGA AAATAATATT**

**17401 CCGGTGCGAC CTGAAGTGGC AAGCGTGTGT GAAATATTAG GCTTTGATCC GTTTGTTTTA**

**17461 GCGTGTGAGG GACGCATTGT TGCTGTTGTT GCCCCTGACA TAAGCGGTAA GGTGCTAGAA**

**17521 CACTGGCGGA GCATTCGAAA CGGGGAGCAG GCCGAACACA TTGGTGTAAT AGTAAAAAAC**

**17581 ACCGAATCAA AAGGGCGCGT GACCATTGTT ACGCCAATGG GAGGAAGACG TTTCATGAAT**

**17641 GAACTCGAAG ATGAGCCGCT TCCAAGGATC TGCTGAGATC CTCTAGAGTC GACCTGCAGG**

**17701 CATGCAAGCT TGGCTGTTTT GGCGGATGAG AGAAGATTTT CAGCCTGATA CAGATTAAAT**

**17761 CAGAACGCAG AAGCGGTCTG ATAAAACAGA ATTTGCCTGG CGGCAGTAGC GCGGTGGTCC**

**17821 CACCTGACCC CATGCCGAAC TCAGAAGTGA AACGCCGTAG CGCCGATGGT AGTGTGGGGT**

**17881 CTCCCCATGC GAGAGTAGGG AACTGCCAGG CATCAAATAA AACGAAAGGC TCAGTCGAAA**

**17941 GACTGGGCCT TTCGTTTTAT CTGTTGTTTG TCGGTGAACG CTCTCCTGAG TAGGACAAAT**

**18001 CCGCCGGGAG CGGATTTGAA CGTTGCGAAG CAACGGCCCG GAGGGTGGCG GGCAGGACGC**

**18061 CCGCCATAAA CTGCCAGGCA TCAAATTAAG CAGAAGGCCA TCCTGACGGA TGGCCTTTTT**

**18121 GCGTTTCTAC AAACTCTTTT GTTTATTTTT CTAAATACAT TCAAATATGT ATCCGCTCAT**

**18181 GGCGGCCCAG ATCCCCGGGT ACCGAGCTCG AATTTCGAGC TTCTGGAGCA GGAAGATGTC**

**18241 GCGGGCATTA GCACCAGCGG TCTGCCAAGC CTCCGCCAGC CGTTGGGTCC CTTCCGCTTG**

**18301 AGCTTTTCCA TCTTCGACGA TACGGGCGGC GGCCCCCCGC GCTTCCGCGA TCGCCCGTTT**

**18361 ACAAGCTGCC TCAGCTGGGG CGATCACATC GGCTTGAAGT TGCTGCTGCA CCTGTTTGAT**

**18421 CCGCTCCTGC TGCACAGGGA GTTCTGCTTG GCTACGAGCG ACTTCGGTAG CAATGTCCGC**

**18481 TTCAGCTTCG GCCACCACCG CTTCGCGCCG CGTCAACGCA TCCTGAATCC GGCGCTCGGC**

**18541 CTCGGCTTGG GCGATCGCTA CATCGCGATC GATCCGACGC AGGGCCGTGA TCTTGTCATT**

**18601 TTCGGCCGTT TGGATCGCAG AGGCAGCCTG GGCATCGGCT TCAGCAATTC GGGCATCTCG**

**18661 CTGCAGATCA GCCCGCTGCT TGCGTCCACT AGCCGAGAGA TAACCGACCT CATCGGAAAT**

**18721 GTTCTGGACT TGCAGCGTAT CGAGGACTAG ACCCAGCTGC TCAAGGTCAT CCTCCGCCTC**

**18781 TTCCAGCAGA CTTTTGGCAA AGGCAATTTT GTCCTCGTTG ATCTGCTCCG GCGTGAGGCT**

**18841 GGCTAAAACA CCACGCAAGT TGCCTTCGAG GGTCTCCTTG GCAATTTGCT CGATTTCCTT**

**18901 ACGGTTTTTG CCAAGCAGCC GCTCGATCGC GTTGTGGATG GTCGGTTCTT CCCCAGCAAT**

**18961 CTTGATATTG GCAACGCCTT CAACAGTCAG GGGAATGCCG CCCTTGGAGA AGGCATTGGA**

**19021 AACGCGCAAC TCAATGATCA TGTTGGTCAG ATCCATGCGG AGCGCTTTTT CCAGCAGAGG**

**19081 TACCCGCAGG CTGCTGCCGC CCTTGACCAA GCGATAGCCA ACTCGGCGGC CATCACTACT**

**19141 GCGGCGACTA CTGCCAGCAA AGATCAAAAT TTCACTGGGT TGGCAGATGT AGTAGAGATT**

**19201 GCGCAGGACT AAGCTGCCAG CCCCGGCGAC TCACCAGTCA CAGAAAAGCA TCTTACGGAT**

**19261 GGCATGACAG TAAGAGAATT ATGCAGTGCT GCCATAACCA TGAGTGATAA CACTGCGGCC**

**19321 AACTTACTTC TGACAACGAT CGGAGGACCG AAGGAGCTAA CCGCTTTTTT GCACAACATG**

**19381 GGGGATCATG TAACTCGCCT TGATCGTTGG GAACCGGAGC TGAATGAAGC CATACCAAAC**

**19441 GACGAGCGTG ACACCACGAT GCCTGCAGCA ATGGCAACAA CGTTGCGCAA ACTATTAACT**

**19501 GGCGAACTAC TTACTCTAGC TTCCCGGCAA CAATTAATAG ACTGGATGGA GGCGGATAAA**

**19561 GTTGCAGGAC CACTTCTGCG CTCGGCCCTT CCGGCTGGCT GGTTTATTGC TGATAAATCT**

**19621 GGAGCCGGTG AGCGTGGGTC TCGCGGTATC ATTGCAGCAC TGGGGCCAGA TGGTAAGCCC**

**19681 TCCCGTATCG TAGTTATCTA CACGACGGGG AGTCAGGCAA CTATGGATGA ACGAAATAGA**

**19741 CAGATCGCTG AGATAGGTGC CTCACTGATT AAGCATTGGT AACTGTCAGA CCAAGTTTAC**

**19801 TCATATATAC TTTAGATTGA TTTAAAACTT CATTTTTAAT TTAAAAGGAT CTAGGTGAAG**

**19861 ATCCTTTTTG ATAATCTCAT GACCAAAATC CCTTAACGTG AGTTTTCGTT CCACTGAGCG**

**19921 TCAGACCCCG TAGAAAAGAT CAAAGGATCT TC**

**//**
